# Function and regulation of the *Caenorhabditis elegans* Rab32 family member GLO-1 in lysosome-related organelle biogenesis

**DOI:** 10.1371/journal.pgen.1007772

**Published:** 2018-11-12

**Authors:** Caitlin Morris, Olivia K. Foster, Simran Handa, Kimberly Peloza, Laura Voss, Hannah Somhegyi, Youli Jian, My Van Vo, Marie Harp, Fiona M. Rambo, Chonglin Yang, Greg J. Hermann

**Affiliations:** 1 Department of Biology, Lewis & Clark College, Portland, Oregon, United States of America; 2 State Key Laboratory of Molecular and Developmental Biology, Institute of Genetics and Developmental Biology, Chinese Academy of Sciences, Beijing, China; University of California San Francisco, UNITED STATES

## Abstract

Cell type-specific modifications of conventional endosomal trafficking pathways lead to the formation of lysosome-related organelles (LROs). *C*. *elegans* gut granules are intestinally restricted LROs that coexist with conventional degradative lysosomes. The formation of gut granules requires the Rab32 family member GLO-1. We show that the loss of *glo-1* leads to the mistrafficking of gut granule proteins but does not significantly alter conventional endolysosome biogenesis. GLO-3 directly binds to CCZ-1 and they both function to promote the gut granule association of GLO-1, strongly suggesting that together, GLO-3 and CCZ-1 activate GLO-1. We found that a point mutation in GLO-1 predicted to spontaneously activate, and function independently of it guanine nucleotide exchange factor (GEF), localizes to gut granules and partially restores gut granule protein localization in *ccz-1(-)* and *glo-3(-)* mutants. CCZ-1 forms a heterodimeric complex with SAND-1(MON1), which does not function in gut granule formation, to activate RAB-7 in trafficking pathways to conventional lysosomes. Therefore, our data suggest a model whereby the function of a Rab GEF can be altered by subunit exchange. *glo-3(-)* mutants, which retain low levels of GLO-3 activity, generate gut granules that lack GLO-1 and improperly accumulate RAB-7 in a SAND-1 dependent process. We show that GLO-1 and GLO-3 restrict the distribution of RAB-7 to conventional endolysosomes, providing insights into the segregation of pathways leading to conventional lysosomes and LROs.

## Author summary

Lysosome-related organelles represent a diverse collection of intracellular compartments that have important physiological functions. In mammals they include melanosomes, key sites of pigment synthesis, and platelet dense granules, which play important roles in blood clotting. Defects in the pathways that direct the formation of these LROs cause Hermansky-Pudlak syndrome, which is characterized by partial albinism and excessive bleeding. Here we describe studies of an LRO found in *Caenorhabditis elegans*, whose formation is mediated by evolutionarily conserved machinery and pathways. Our work supports a novel model for how a key player in LRO formation, the Rab32 orthologue GLO-1, is regulated and functions in *C*. *elegans*.

## Introduction

*Caenorhabditis elegans* gut granules are lysosome related organelles (LROs) [1], cell type-restricted compartments with diverse functions that share characteristics with conventional lysosomes [2]. These conspicuous intestine-specific compartments contain birefringent and autofluorescent material [3–5]. Gut granules function in lipid transport [6], metabolism [7, 8], and signaling [9], as well as the storage and detoxification of metals and xenobiotics [10–12]. Gut granules coexist with conventional lysosomes and are not the major degradative compartments within *C*. *elegans* intestinal cells [1, 5, 13, 14].

LRO biogenesis is mediated by evolutionarily conserved pathways that divert cargo away from conventional endosomes toward LROs [15, 16]. Defects in these pathways cause Hermansky-Pudlak syndrome, a human condition characterized by a lack of dense granules and malformed melanosomes, LROs found within platelets and melanocytes, respectively [17]. Screens for *C*. *elegans* mutants that disrupt gut granule biogenesis have identified conserved factors that promote LRO biogenesis. Similar to many mammalian LROs, gut granule formation requires the BLOC-1 [18], HOPS [19], and AP-3 complexes [1], LYST [20], and the Rab32 family member GLO-1 [1].

Rab32 family members have a conserved function in LRO biogenesis and are one of 20 Rabs hypothesized to have been present in the last eukaryotic common ancestor [1, 21–28]. The reversible association of Rabs with membranes, where they regulate key cargo trafficking events, is in large part mediated by guanine nucleotide exchange factors (GEFs) [29–32]. In mammals, the BLOC-3 GEF activates and localizes the Rab32 family members Rab32 and Rab38 [33]. BLOC-3 is a heterodimeric complex composed of two proteins, HPS1 and HPS4 [34–36]. Despite the presence of a Rab32 family member, HPS1 and HPS4 are not obviously conserved in *C*. *elegans* [37], raising the question of how GLO-1 is activated and localized.

HPS1 and HPS4 have weak homology to CCZ-1 and SAND-1(MON1), respectively [33, 38, 39], which form a heterodimeric GEF activating Rab7 in the conventional endolysosomal trafficking pathway [40–43]. Rab7 and Rab32 family members function in endosomal trafficking pathways, are closely related, and might share regulatory factors or effectors [22]. We recently found that *ccz-1(-)* mutants lack gut granules, whereas *rab-7(-)* mutants have only a minor defect in gut granule protein localization, and *sand-1* is dispensable for gut granule biogenesis [19]. These phenotypes indicate an unexpected role for CCZ-1 in LRO biogenesis that does not involve its known interactions with SAND-1(MON1) or its regulation of RAB-7. Given the Rab GEF activity of CCZ-1 and the homology of Rab32 family members with Rab7, CCZ-1 might function with a different protein to activate and localize GLO-1. Our recent work identified weak homology between GLO-3 and HPS1 and placed GLO-3, like CCZ-1, function upstream of GLO-1 activation, providing an initial indication that this interacting factor might be GLO-3 [19].

Here we present the results of studies analyzing the physical association of GLO-3 and CCZ-1 and the functional relationships between CCZ-1 and GLO-3 in GLO-1 localization and gut granule and conventional endolysosome biogenesis. Our results show that CCZ-1 and GLO-3 function to localize GLO-1 to LRO membranes and promote gut granule formation. However, GLO-3 and GLO-1 do not function in endolysosome biogenesis like CCZ-1 and instead act to restrict RAB-7 to conventional endolysosomes.

## Results

### GLO-3 and CCZ-1 physically interact

**Fig 1:**
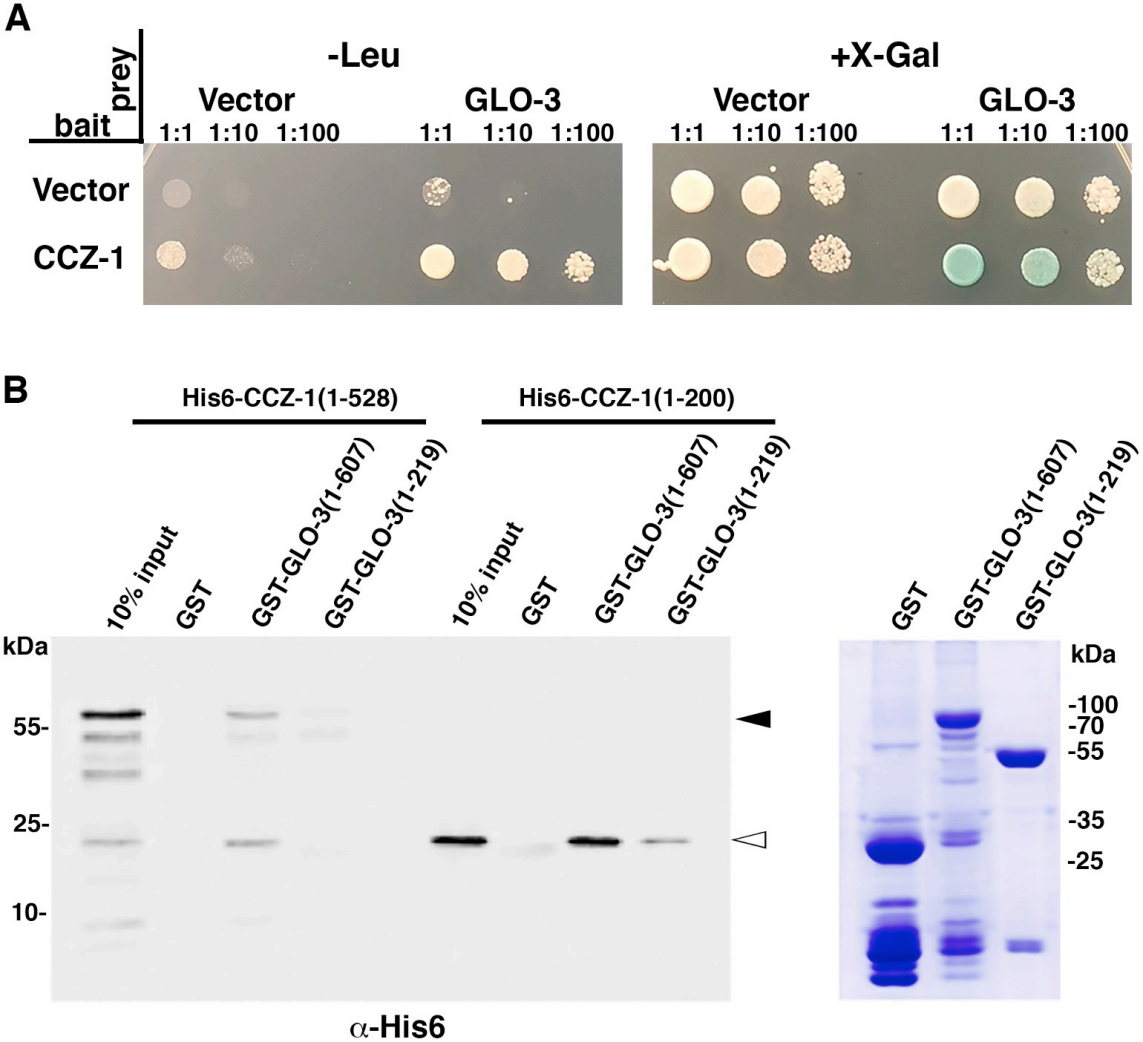
GLO-3 and CCZ-1 physically interact. (A) Testing for interactions between full length CCZ-1 and GLO-3 using the yeast 2-hybrid system. Yeast cells were cotransformed with plasmids encoding the LexA DNA binding domain (bait) and B42 transcription activation domain (prey) fusions. Serial dilutions were spotted on the indicated medias. Growth on–Leu or production of a blue color on +X-Gal media is indicative of a protein-protein interaction. (B) Glutathione beads loaded with recombinant GST (MW 26kDa), GST-GLO-3 (full length, amino acids 1–607, MW 93kDa), or GST-GLO-3 (amino acids 1–219, MW 50kDa) (right panel, Coomassie stained gel) were incubated with purified His6-tagged CCZ-1 (full length, amino acids 1–528) or His6-tagged CCZ-1 (amino acids 1–200) and then were extensively washed to remove unbound protein. Bound proteins were separated by SDS/PAGE and visualized by Western blot with anti-His6 antibodies. The white arrowhead denotes His6-CCZ-1 (full length) and the black arrowhead denotes His6-CCZ-1 (amino acids 1–200).

To determine if GLO-3 can physically interact with CCZ-1, we screened for interactions between the two full-length proteins using the yeast two-hybrid system. When used as bait, CCZ-1 interacted with the GLO-3 prey, promoting the expression of both LEU2 and LacZ reporters (Fig 1A).

We confirmed the interaction between GLO-3 and CCZ-1 by expressing the proteins in *E*. *coli* and using a glutathione S-transferase (GST)-pull down assay. Full length GST-GLO-3 pulled down both full length CCZ-1 and the CCZ-1(1–200) amino terminal region (Fig 1B). GLO-3(1–219) was able to pull down CCZ-1(1–200), albeit not as strongly as full length GLO-3 (Fig 1B). The GST moiety attached to GLO-3 did not significantly interact with either form of CCZ-1 (Fig 1B). Taken together, these results show that GLO-3 and CCZ-1 directly interact and that the amino-terminal domain of CCZ-1, which contains a longin domain [40], acts as a binding interface between the two proteins.

### GLO-1 and GLO-3 promote protein localization to gut granules and not lysosomes

We analyzed whether the effects of mutations in *glo-1* and *glo-3* on gut granule and endolysosome biogenesis resembled the loss of *ccz-1* function. The *glo-1(zu437)* allele used in our studies completely lacks GLO-1 activity [1]. We have isolated a *glo-3* allelic series spanning four phenotypic classes (I-IV) [44], ranging from the least severe, a newly identified class IV *glo-3(gk582755)* missense allele GLO-3(N279K) that causes a moderate reduction in gut granule numbers, to the strongest class I alleles, represented by *glo-3(kx94)*, which lack birefringent gut granules in embryos but retain a few autofluorescent gut granules in adults (Tables 1 and 2). Nearly all of the *glo-3* alleles are premature stop codons and their location in *glo-3* does not correlate with their phenotypic severity [44]. We note that the two class I alleles cause amber stop codons, while the weaker class II and class III alleles cause ochre or opal stop codons [44]. In *C*. *elegans*, opal and ochre stop codons can occasionally be read through during translation, while amber stop codons cannot [45], suggesting that the class II and III alleles produce differing amounts of full length GLO-3. CRISPR-Cas9 was used to delete the entire *glo-3* coding sequence and the resulting *glo-3(syb272)* allele was phenotypically indistinguishable from class I mutants (S1 Fig and Tables 1 and 2), strongly suggesting that the *glo-3(kx94)* allele used in this work represents a null allele.

**Table 1:** Gut granules in embryos expressing GLO-1 point mutations.

Genotype	% of embryos with the specified number of birefringent granules in intestinal cells				
	0	1–20	21–50	>50	*n*
Wild type	0	0	0	100	292
Wild type + *gfp*::*glo-1(+)*	0	0	8	92	103
Wild type + *gfp*::*glo-1(Q71L)*	0	0	0	100	101
Wild type + *gfp*::*glo-1(T25N)*	0	0	1	99	101
*glo-1(zu437)*	96	4	0	0	187
*glo-1(zu437) + gfp*::*glo-1(+)*	0	0	5	95	163
*glo-1(zu437) + gfp*::*glo-1(Q71L)*	0	0	0	100	101
*glo-1(zu437) + gfp*::*glo-1(T25N)*	100	0	0	0	100
*glo-1(zu437) + gfp*::*glo-1(D132A)*	0	3	31	66	67
*glo-1(zu437) + gfp*::*glo-1(I133F)*	0	0	6	94	20
*glo-3(kx94)*	87	13	0	0	170
*glo-3(kx94) + gfp*::*glo-1(+)*	72	28	0	0	64
*glo-3(kx94) + gfp*::*glo-1(D132A)*	0	11	40	49	80
*glo-3(kx94) + gfp*::*glo-1(I133F)*	0	50	50	0	22
*glo-3(kx38)*	0	89	11	0	64
*glo-3(kx38) + gfp*::*glo-1(+)*	0	95	5	0	20
*glo-3(kx38) + gfp*::*glo-1(D132A)*	0	0	5	95	21
*glo-3(kx38) + gfp*::*glo-1(I133F)*	0	12	56	32	32
*glo-3(zu446)*	11	89	0	0	64
*glo-3(zu446) + gfp*::*glo-1(+)*	14	76	10	0	22
*glo-3(zu446) + gfp*::*glo-1(D132A)*	0	0	2	98	41
*glo-3(syb272)*	95	5	0	0	63
*glo-3(gk582755)*	0	16	84	0	146
*glo-1(zu437) glo-3(zu446)*	95	5	0	0	40
*glo-1(zu437) glo-3(zu446) + gfp*::*glo-1(+)*	57	33	10	0	20
*glo-1(zu437) glo-3(zu446) + gfp*::*glo-1(D132A)*	0	0	0	100	20
*ccz-1(ok2182)*	65	35	0	0	86
*ccz-1(ok2182) + gfp*::*glo-1(+)*	72	28	0	0	60
*ccz-1(ok2182) + gfp*::*glo-1(D132A)*	0	2	12	86	59
*apt-7(tm920)*	25	40	35	0	69
*apt-7(tm920) + gfp*::*glo-1(+)*	90	10	0	0	20
*apt-7(tm920) + gfp*::*glo-1(D132A)*	71	29	0	0	42
*snpn-1(tm1892)*	100	0	0	0	39
*snpn-1 (tm1892) + gfp*::*glo-1(+)*	100	0	0	0	20
*snpn-1 (tm1892) + gfp*::*glo-1(D132A)*	100	0	0	0	20
*vps-18(tm1125)*	100	0	0	0	38
*vps-18(tm1125) + gfp*::*glo-1(+)*	100	0	0	0	21
*vps-18(tm1125) + gfp*::*glo-1(D132A)*	100	0	0	0	24

All strains were grown at 22°C. Three-fold and later stage embryos were analyzed using polarization microscopy and scored for the presence and number of birefringent granules in the intestine. The expression of *gfp*::*glo-1* was assayed with fluorescence microscopy.

**Table 2:** Autofluorescent gut granules in adults expressing GLO-1 point mutations.

Genotype	% of animals with the specified number of autofluorescent organelles in the intestine						*n*
	0	1–20	21–50	51–100	101–200	>200	
Wild type	0	0	0	0	0	100	100
Wild type *+ gfp*::*glo-1(+)*	0	0	0	0	0	100	20
Wild type *+ gfp*::*glo-1(Q71L)*	0	0	0	0	0	100	20
Wild type *+ gfp*::*glo-1(T25N)*	0	0	0	0	0	100	20
*glo-1(zu437)*	94	6	0	0	0	0	85
*glo-1(zu437) + gfp*::*glo-1(+)*	0	0	0	0	10	90	62
*glo-1(zu437) + gfp*::*glo-1(Q71L)*	0	0	0	0	0	100	20
*glo-1(zu437) + gfp*::*glo-1(T25N)*	90	10	0	0	0	0	20
*glo-1(zu437) + gfp*::*glo-1(D132A)*	0	0	0	0	17	83	41
*glo-1(zu437) + gfp*::*glo-1(I133F)*	0	0	0	0	8	92	30
*glo-3(kx94)*	32	55	12	1	0	0	85
*glo-3(kx94) + gfp*::*glo-1(+)*	33	67	0	0	0	0	45
*glo-3(kx94) + gfp*::*glo-1(D132A)*	0	0	0	0	0	100	40
*glo-3(kx94) + gfp*::*glo-1(I133F)*	0	0	0	55	36	9	26
*glo-3(kx38)*	0	7	59	31	3	0	70
*glo-3(kx38) + gfp*::*glo-1(+)*	0	0	48	52	0	0	21
*glo-3(kx38) + gfp*::*glo-1(D132A)*	0	0	0	0	23	87	23
*glo-3(kx38) + gfp*::*glo-1(I133F)*	0	0	0	1	37	62	27
*glo-3(zu446)*	0	44	56	0	0	0	45
*glo-3(zu446) + gfp*::*glo-1(+)*	0	50	50	0	0	0	20
*glo-3(zu446) + gfp*::*glo-1(D132A)*	0	0	0	0	10	90	20
*glo-3(syb272)*	0	23	73	3	0	0	30
*glo-3(gk582755)*	0	0	0	60	23	17	40
*glo-1(zu437) glo-3(zu446)*	35	65	0	0	0	0	46
*glo-1(zu437) glo-3(zu446) + gfp*::*glo-1(+)*	4	50	36	0	0	0	22
*glo-1(zu437) glo-3(zu446) + gfp*::*glo-1(D132A)*	0	0	0	0	0	100	20
*ccz-1(ok2182)*	71	23	6	0	0	0	64
*ccz-1(ok2182) + gfp*::*glo-1(+)*	50	47	3	0	0	0	40
*ccz-1(ok2182) + gfp*::*glo-1(D132A)*	0	0	0	0	29	71	34
*apt-7(tm920)*	12	71	17	0	0	0	42
*apt-7(tm920) + gfp*::*glo-1(+)*	40	60	0	0	0	0	25
*apt-7(tm920) + gfp*::*glo-1(D132A)*	0	77	23	0	0	0	22
*snpn-1(tm1892)*	78	22	0	0	0	0	40
*snpn-1 (tm1892) + gfp*::*glo-1(+)*	100	0	0	0	0	0	26
*snpn-1 (tm1892) + gfp*::*glo-1(D132A)*	45	55	0	0	0	0	22
*vps-18(tm1125)*	0	0	0	0	0	100	43
*vps-18(tm1125) + gfp*::*glo-1(+)*	0	0	0	0	0	100	20
*vps-18(tm1125) + gfp*::*glo-1(D132A)*	0	0	0	0	0	100	20

All strains were grown 22°C. Adults were analyzed using fluorescence microscopy with a rhodamine filter to score the number of autofluorescent organelles within the intestine located posterior to the vulva and a FITC filter set to assess the expression of GFP::GLO-1.

**Fig 2:**
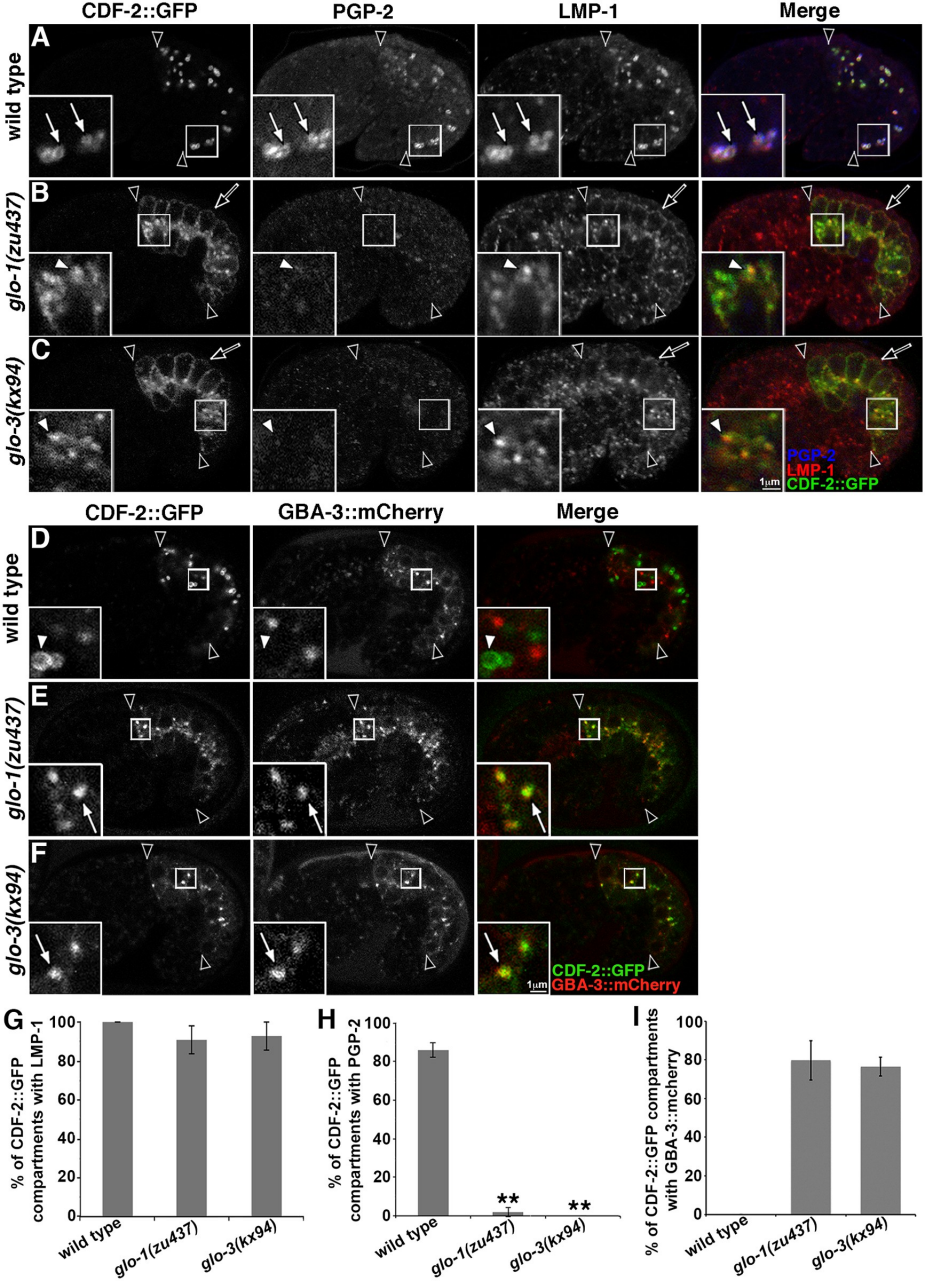
Gut granule protein localization in *glo-1(-)* and *glo-3(-)*. (A-C) 1.5 fold stage embryos expressing CDF-2::GFP were stained with anti-PGP-2 and anti-LMP-1 antibodies and imaged with confocal microscopy. The three proteins colocalized at gut granules in wild type (white arrows within insets). *glo-1(-)* and *glo-3(-)* mutants mislocalized CDF-2::GFP and LMP-1 to the cell membrane (black arrows), lacked PGP-2 staining (white arrowheads within insets), and CDF-2::GFP marked organelles contained LMP-1. (D-F) The localization of CDF-2::GFP and lysosomal hydrolase GBA-3::mCherry were analyzed in living 1.5 fold stage embryos with confocal microscopy. GBA-3::mCherry did not localize to CDF-2::GFP marked compartments in wild type (white arrowheads in insets), however it often did (white arrows within insets) in *glo-1* and *glo-3* mutants. (G-H) For each genotype, 20 randomly selected CDF-2::GFP containing intestinal compartments in 5 different embryos were scored for the presence of LMP-1 or PGP-2 signals. (I) For each genotype, 25 randomly selected CDF-2::GFP containing intestinal compartments in 5 different 1.5-fold stage embryos were scored for the presence of GBA-3::mCherry. In all images, a single optical section is shown and black arrowheads flank the intestine. Embryos are 50μm in length. In all graphs, the mean is plotted and error bars represent the 95% confidence limit. A one way ANOVA comparing each mutant to wild type was used to calculate p-values (** represents p ≤ 0.001).

In prior work, we showed that disrupting the function of *glo-1* or *glo-3* leads to significant reductions in autofluorescent and birefringent gut granules (Tables 1 and 2) [1, 19]. To determine if this is associated with defects in protein localization in *glo-1(-)* and *glo-3(-)* mutants, we analyzed the steady state distribution of the gut granule transmembrane proteins CDF-2, which functions as a Zn transporter [46], the ABC transporter PGP-2 [47], and the Lamp1 homologue LMP-1 [48]. CDF-2 and PGP-2 are restricted to gut granules, while LMP-1 is associated with both gut granules and conventional lysosomes [13]. To minimize indirect effects, we carried out our analyses in embryonic intestinal cells soon after gut granules are first generated [3]. In *glo-1(zu437)* and *glo-3(kx94)* mutants, the distribution of CDF-2::GFP and LMP-1 was dramatically altered (Fig 2B and 2C). Strikingly, both were mislocalized to the intestinal cell membrane (Fig 2B and 2C). While the extensive colocalization of CDF-2::GFP and LMP-1 was not disrupted in these mutants, they were not located on organelles that resembled gut granules (Fig 2B, 2C and 2G). Instead, the morphology and position of the compartments containing these markers were similar to conventional lysosomes. To examine whether CDF-2::GFP was being mislocalized to lysosomes, we analyzed the distribution of CDF-2::GFP relative to an mCherry tagged form of GBA-3, a glucosylceramidase localized to degradative lysosomes that when disrupted in humans causes Gaucher disease [13, 49, 50]. We found that CDF-2::GFP was mislocalized to conventional lysosomes in *glo-1(-)* and *glo-3(-)* mutants (Fig 2D, 2F and 2I), explaining why the colocalization of CDF-2::GFP and LMP-1 was not altered in these mutants. PGP-2 was lacking in both *glo-1(-)* and *glo-3(-)* mutants (Fig 2B, 2C and 2H), possibly due to its lysosomal mistargeting and degradation. These results indicate that GLO-1 and GLO-3 both play essential roles in the routing of proteins to gut granules, similar to what we have previously seen for CCZ-1 [19].

**Fig 3:**
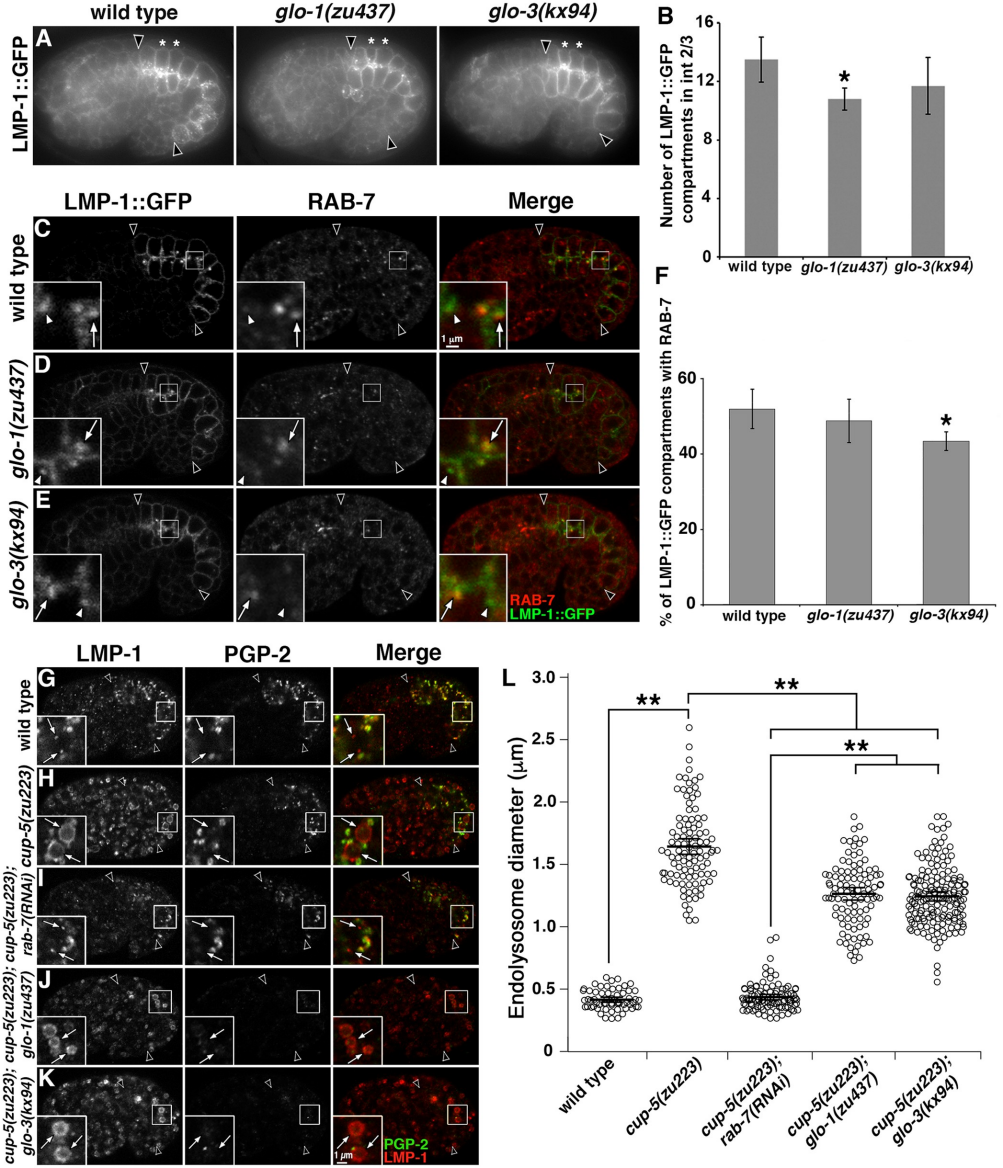
Endolysosomes in *glo-1(-)* and *glo-3(-)*. (A) Living 1.5 fold stage embryos expressing LMP-1::GFP were imaged with wide field microscopy. (B) The total number of LMP-1::GFP compartments was quantified in the four cells that compose Int2 and Int3 (marked with white asterisks in A) in ten 1.5-fold stage embryos of each genotype. (C-E) 1.5-fold stage embryos expressing LMP-1::GFP were stained with anti-RAB-7 antibodies and imaged with confocal microscopy. RAB-7 associates with many (white arrows in insets), but not all (white arrowheads in insets), LMP-1::GFP marked compartments in the three strains. (F) Colocalization was assessed in 5 embryos of each genotype by randomly selecting 17 to 25 LMP-1::GFP organelles in each embryo and scoring for the RAB-7 signal. (G-K) 1.5 fold stage embryos were stained with anti-LMP-1 and anti-PGP-2 antibodies and imaged with confocal microscopy. Endolysosomes were marked by LMP-1 and lacked PGP-2 (white arrows in insets). The enlarged endolysosomes in *cup-5* mutants were reduced to wild-type size when exposed to *rab-7(RNAi)*. Enlarged endolysosomes remained when *glo* mutations were introduced into a *cup-5(-)* background. (L) For each genotype, the diameters of 20 randomly selected LMP-1 organelles that lacked PGP-2 were measured in 5–9 1.5 fold stage embryos. In all images, a single optical section is shown and black arrowheads flank the intestine. In all graphs, the mean is plotted and error bars represent the 95% confidence limit. In B and F a one way ANOVA comparing each mutant to wild type was used to calculate p-values. In L, a one way ANOVA followed by a Tukey-Kramer post-hoc test was used to compare different genotypes (* represents p ≤ 0.05 and ** represents p ≤ 0.001).

We next addressed whether GLO-1 and GLO-3 function in the formation of lysosomes. Gut granules and conventional lysosomes are distinct and co-exist in embryonic intestinal cells [13]. Some factors that mediate gut granule biogenesis, including CCZ-1, also function in lysosome biogenesis and disrupting their activity causes a significant increase in the number of endolysosomes marked by LMP-1::GFP [19]. LMP-1::GFP, like endogenous LMP-1, is localized to conventional lysosomes, however the addition of GFP to its cytoplasmic carboxyl-terminus disrupts it’s sorting, causing its loss from gut granules and enrichment at the cell membrane [13]. In *glo-1(-)* mutants there was a slight decrease in the number of LMP-1::GFP compartments and *glo-3(-)* mutants were unchanged relative to wild type (Fig 3A and 3B), which is very different than what we see in *ccz-1(-)* mutants, where the number is increased two-fold [19].

RAB-7 dynamically localizes to endolysosomes as they mature and defective trafficking to conventional lysosomes significantly alters the colocalization of RAB-7 and LMP-1::GFP [19, 51]. Approximately 50% of LMP-1::GFP compartments were labeled by RAB-7 in wild type, a level of colocalization that was not markedly different in *glo-1(-)* and *glo-3(-)* mutants (Fig 3C–3F).

In *C*. *elegans* coelomocytes, decreased trafficking to lysosomes suppresses the enlargement of lysosomes caused by mutations in CUP-5 [52]. CUP-5 is orthologous to human TRPML1 [53, 54], which is mutated in type IV mucolipidosis, and mediates the formation of lysosomes from endosomal-lysosomal hybrid compartments [55]. LMP-1 containing endolysosomes were enlarged in *cup-5(zu223)* embryonic intestinal cells (Fig 3G, 3H and 3L). Moreover, *rab-7(RNAi)* disrupts trafficking to lysosomes and reduced *cup-5(-)* endolysosomes back to wild-type size, validating the assay in this cell type (Fig 3I and 3L). In contrast, endolysosomes in *cup-5(-); glo-1(-)* and *cup-5(-); glo-3(-)* double mutants remained significantly enlarged, albeit not quite as large as in *cup-5(-)* (Fig 3J–3L). Together these observations point to GLO-1 and GLO-3 having an essential role in localizing gut granule proteins and a minor, if any, role in trafficking to conventional lysosomes.

### GLO-1 and GLO-3 impact CDF-2::GFP and LMP-1 protein localization differently than CCZ-1

**Fig 4:**
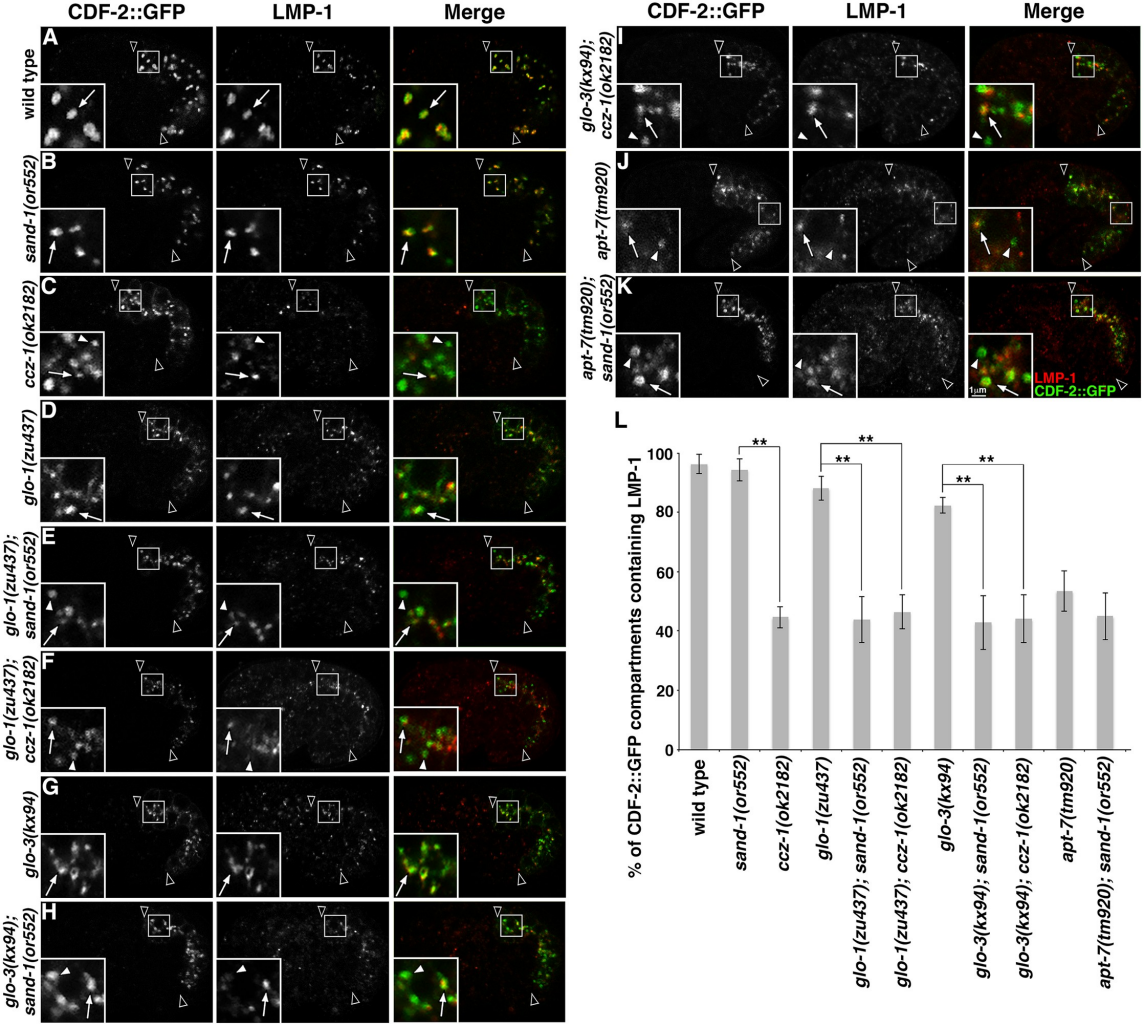
Gut granule protein localization in double mutants with *sand-1(-)* and *ccz-1(-)*. (A-K) Signals from anti-LMP-1 antibodies and CDF-2::GFP in 1.5-fold stage embryos were acquired by confocal microscopy. In insets, CDF-2::GFP labeled organelles containing LMP-1 are marked with white arrows and those lacking LMP-1 are marked with white arrowheads. Single optical sections are shown, black arrowheads flank the intestine. (L) For each genotype, at least 25 randomly selected CDF-2::GFP organelles in 5 different embryos were scored for the presence of anti-LMP-1 signals. The mean is plotted and error bars represent the 95% confidence limit. A one way ANOVA comparing mutants was used to calculate p-values (** represents p ≤ 0.001).

In *glo-1(-)* and *glo-3(-)* mutants, the mislocalization of the gut granule protein CDF-2::GFP to conventional lysosomes led to a high level of colocalization between CDF-2::GFP and LMP-1, which marks lysosomes (Figs 2, 4D, 4G and 4L). In contrast, only 50% of CDF-2::GFP compartments were marked by LMP-1 in *ccz-1(-)* mutants (Fig 4C and 4L), revealing a significant phenotypic difference in gut granule protein localization between *ccz-1(-)* and the *glo-1(-)* and *glo-3(-)* mutants. In double mutants, the colocalization phenotype of *ccz-1(-)* was epistatic to *glo-1(-)* and *glo-3(-)* (Fig 4F, 4I and 4L). While it is currently unclear whether *ccz-1(-)* impacts the colocalization of CDF-2::GFP and LMP-1 by mislocalizing CDF-2::GFP, LMP-1, or both proteins, the data indicate that CCZ-1 has functions in protein localization distinct from GLO-1 and GLO-3.

We investigated whether *sand-1(-)* mutants disrupt the colocalization of CDF-2::GFP and LMP-1 similarly to *ccz-1(-)*, due to the well-established role of the *C*. *elegans* CCZ-1/SAND-1(MON1) complex in the biogenesis of late endosomes and trafficking to conventional lysosomes [42, 56, 57]. In *sand-1(-)* single mutants, the colocalization of CDF-2::GFP with LMP-1 was not obviously different than wild type (Fig 4B and 4L). However, gut granule biogenesis is not disrupted in *sand-1(-)* mutants like it is in *ccz-1(-)* mutants [19]. When *sand-1(-)* was combined with *glo-1(-)* or *glo-3(-)* mutants the colocalization of CDF-2::GFP and LMP-1 was indistinguishable from that of *ccz-1(-)* single mutants (Fig 4B, 4E, 4H and 4L). This effect is consistent with SAND-1 functioning only in localizing proteins to endolysosomes, GLO-1 and GLO-3 functioning only in gut granule protein localization, and CCZ-1 functioning in both processes. In support of this interpretation, disrupting the function of *apt-7*, which encodes a subunit of the AP-3 complex that functions in trafficking to both LROs and conventional lysosomes [58], altered the colocalization of CDF-2::GFP with LMP-1 similar to *ccz-1(-)*, and the addition of *sand-1(-)* did not modify its effects (Fig 4J–4L).

### GLO-1 functions as a Rab GTPase to promote gut granule biogenesis

**Fig 5:**
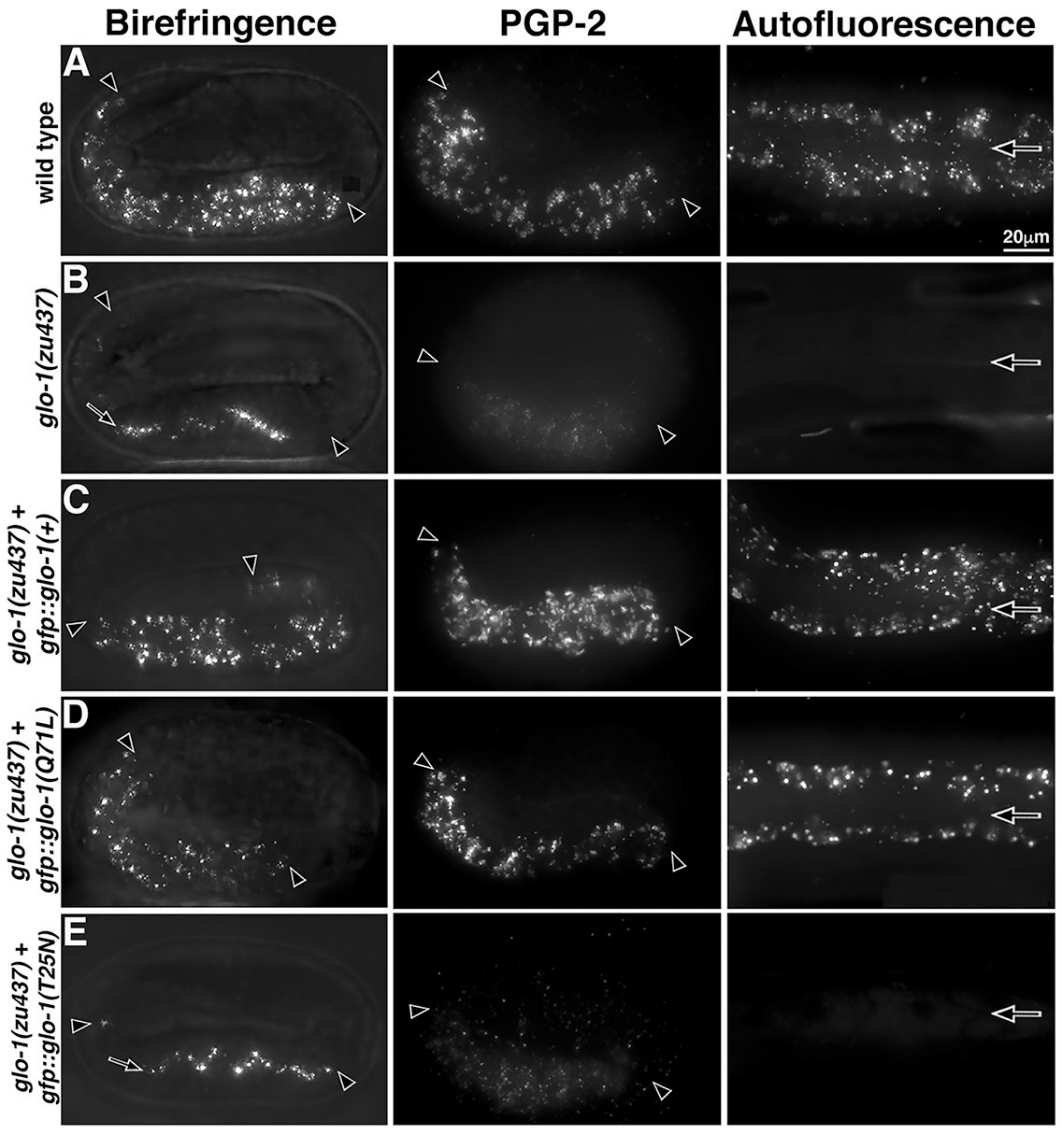
Effects of GLO-1 GTPase activating and inhibitory mutations on gut granule biogenesis. Living pretzel stage embryos were analyzed with polarization microscopy to detect birefringent material and fixed pretzel stage embryos were stained with anti-PGP-2 antibodies. Adults were analyzed for autofluorescent compartments. (A-B) Unlike wild type, *glo-1(-)* mutants lacked birefringent material in intestinal cells, misaccumulating it within the intestinal lumen (black arrow), lacked PGP-2 marked organelles, and lacked autofluorescent gut granules. (C-E) Whereas *glo-1(+)* wild-type and *glo-1(Q71L)* mutant transgenes restored birefringent, PGP-2-containing, and autofluorescent gut granules when expressed in *glo-1(-)* mutants, the *glo-1(T25N)* mutant did not. All imaging was carried out with wide field microscopy. Maximum intensity projections spanning the entire width of the intestine are shown. Embryos are 50μm in length. Black arrowheads flank the intestine in embryos and black arrows mark the lumen within adults.

GLO-1 is a Rab32 family member and the conservation of G-motifs suggests that it functions as a GTPase [21, 22]. To determine if GTP binding is important for the activity of GLO-1 *in vivo* we expressed GLO-1 point mutants in *glo-1(zu437)* animals. Whereas GFP tagged GLO-1(+) restored gut granules in *glo-1(zu437)* (Fig 5A–5C), expression of GFP::GLO-1(T25N), which is predicted to disrupt GTP but not GDP binding [59], did not rescue the loss of autofluorescent, birefringent, and PGP-2 marked gut granules (Fig 5E and Tables 1 and 2). Expression of GFP::GLO-1(Q71L), which is predicted to lack GTP hydrolysis and maintain an active GTP-bound conformation [59], was able to functionally replace GLO-1(+) (Fig 5D and Tables 1 and 2). Neither GLO-1 point mutant dominantly disrupted gut granule biogenesis (Tables 1 and 2). The different mutant effects suggest that the GTP bound form of GLO-1 is active in promoting gut granule biogenesis.

### GLO-3 and CCZ-1 act upstream of GLO-1

**Fig 6:**
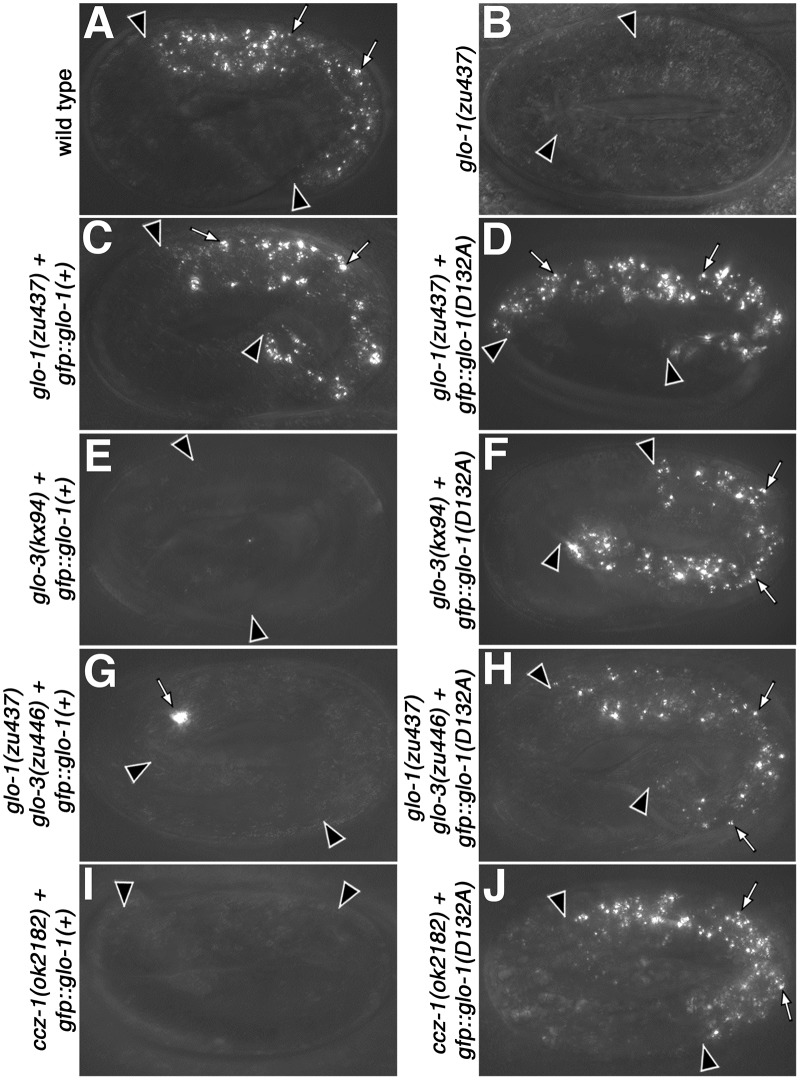
Activity of GLO-1(D132A) in the formation of birefringent gut granules. (A-J) Pretzel stage embryos were analyzed with polarization microscopy to detect birefringent material. (C, E, G, I) *gfp*::*glo-1(+)* restored birefringent gut granules (white arrows) in *glo-1(-)* embryos, but not other *glo* mutants. (D, F, H, J) *gfp*::*glo-1(D132A)* promoted the formation of birefringent gut granules in *glo-1(-)*, *glo-3(-)*, and *ccz-1(-)* embryos. Wide-field maximum intensity projections spanning the entire width of the intestine are shown. Embryos are 50μm in length. Black arrowheads flank the intestine.

Mutations in the Rab G4 motif can weaken its affinity for guanine nucleotides leading to increased rates of nucleotide exchange that can bypass the requirement of a Rab for its corresponding GEF [41, 60, 61]. GLO-1(D132A) and GLO-1(I133F) G4 motif mutations restore autofluorescent compartments in *ccz-1(-)* and *glo-3(-)* mutant adults [19], suggesting that spontaneous nucleotide exchange bypasses the requirement for CCZ-1 or GLO-3 in gut granule biogenesis. The *vha-6* promoter used in our prior study initiates expression late in embryogenesis [62, 63]. To assess the effects of the GLO-1 G4 mutants at a stage when we can rigorously assess the biogenesis of gut granules using multiple organelle markers, we placed the point mutants under control of the *glo-1* promoter, which leads to earlier intestinal expression. When introduced into *glo-1(zu437)* mutants, both GLO-1 G4 mutants restored birefringent compartments in embryonic intestinal cells and autofluorescent intestinal organelles in adults (Fig 6A–6D and Tables 1 and 2). We focused our analysis on GLO-1(D132A) as it had the strongest rescuing activity (Tables 1 and 2). GLO-1(D132A) restored birefringent and autofluorescent granules in *glo-3(-)* and *ccz-1(-)* mutants, but did not suppress the loss of these organelles in AP-3, BLOC-1, or HOPS mutants (Fig 6F and 6J and Tables 1 and 2). Expression of GLO-1(+) only restored birefringent and autofluorescent organelles in *glo-1(-)* mutants (Fig 6C, 6E and 6I and Tables 1 and 2). Therefore, GLO-3 and CCZ-1 likely function upstream of GLO-1 in the formation of gut granules.

In the *ccz-1(-)* and *glo-3(-)* mutant strains where gut granule biogenesis was rescued by GLO-1(D132A), endogenous wild-type *glo-1(+)* is also present. GLO-1(D132A) was introduced into a *glo-1(-) glo-3(-)* double mutant where it fully restored autofluorescent and birefringent compartments (Fig 6H and Tables 1 and 2). This result indicates that the restoration of gut granules in *glo-3(-)* is mediated by GLO-1(D132A) and not by endogenous *glo-1(+)*.

We investigated whether GLO-1(D132A) restored gut granule protein localization in *glo-1(-)*, *glo-3(-)*, and *ccz-1(-)* mutants. For these experiments, we quantified the colocalization of proteins using SQUASSH image analysis software that deconvolves, segments, and calculates the overlapping area between two fluorescence signals in three dimensions [64]. This software enabled a comprehensive, high throughput, and quantitative approach for identifying and measuring the area of overlap between two different organelle markers within the entire embryonic intestine. The output C_size_(marker 1_marker2_/marker 1) describes the proportion of marker 1’s area that contains marker 2.

**Fig 7:**
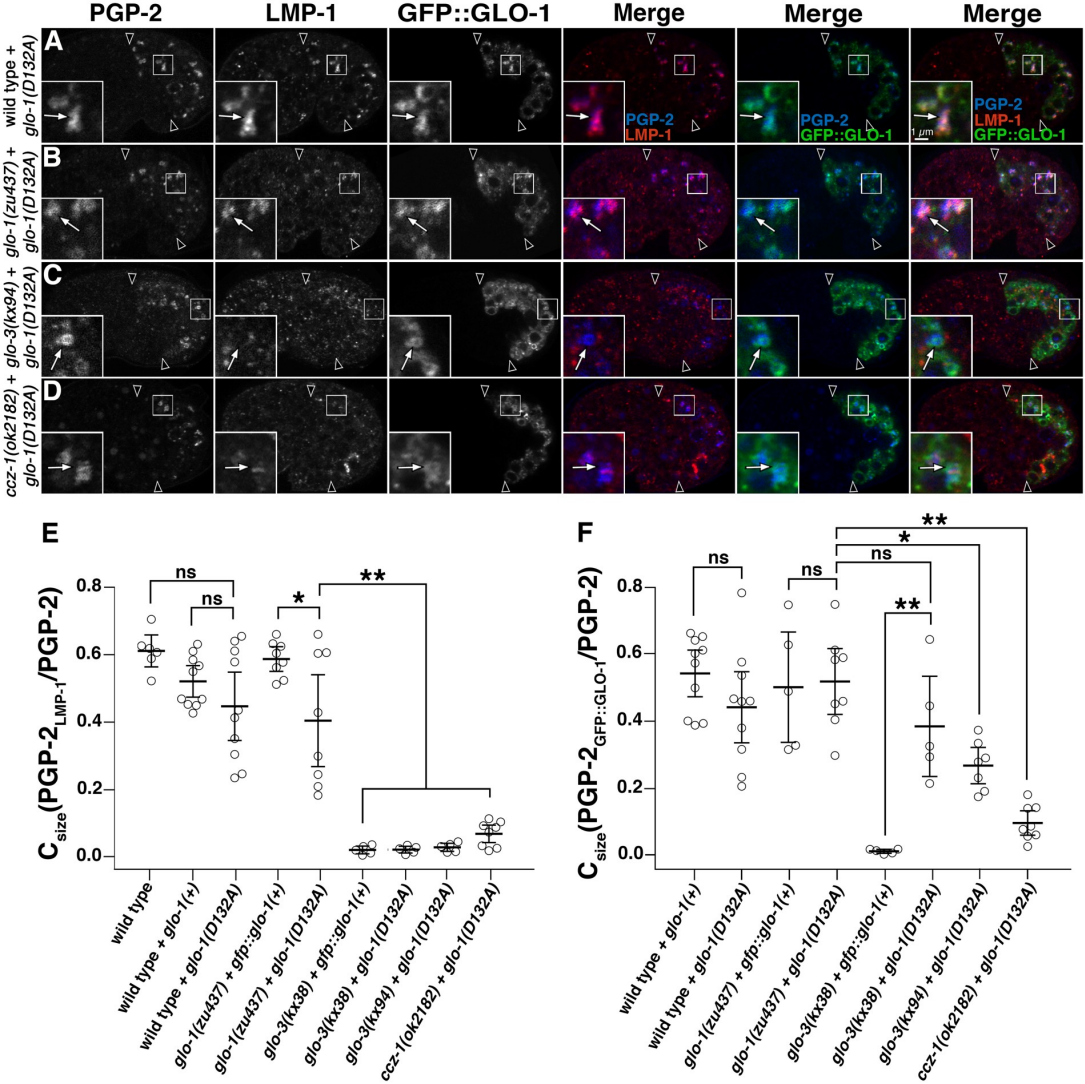
Activity of GLO-1(D132A) in gut granule protein localization. (A-D) 1.5-fold stage embryos expressing GFP::GLO-1(D132A) were stained with antibodies to PGP-2 and LMP-1. Embryos were imaged with confocal microscopy, single optical sections are shown, and white arrows in the insets label organelles containing the gut granule protein PGP-2. Black arrowheads flank the intestine. (E-F) SQUASSH software was used to calculate C_size_(PGP-2_LMP-1 or GFP::GLO-1_/PGP-2), which represents the proportion of total PGP-2 area in each embryo that also contained LMP-1 (E) or GLO-1::GFP (F) signals. Each data point represents the C_size_ of an individual embryo and at least 5 embryos of each genotype were scored. The mean level of colocalization per embryo is plotted, bars represent the 95% confidence intervals, and** indicates p≤0.005, * indicates p≤0.05, and ns indicates p>0.05, by one way ANOVA followed by a Tukey-Kramer test.

PGP-2 labeled organelles were lacking in *glo-1(-)*, *glo-3(-)*, and *ccz-1(-)* mutant embryos (Fig 2B and 2C). Expression of GLO-1(+) or GLO-1(D132A) in *glo-1(-)* embryos restored gut granules containing PGP-2 and LMP-1 (Fig 7B and 7E and S2 Fig). We note that GLO-1(D132A) did not always support the high level of colocalization seen between these markers when GLO-1(+) was expressed (Fig 7B and 7E and S2 Fig). Consistent with our observations of other gut granule markers (Tables 1 and 2), expression of GLO-1(+) in *glo-3(-)* or *ccz-1(-)* mutants did not restore PGP-2 compartments (S2 Fig). In contrast, GLO-1(D132A) robustly supported the formation of PGP-2 marked organelles in both mutants (Fig 7C, 7D and 7E). The rescuing activity of GLO-1(D132A) was not complete however, as these compartments lacked LMP-1 (Fig 7C, 7D and 7E). Therefore, while GLO-1(D132A) can substitute for much of the activity of *glo-3(+)* and *ccz-1(+)* in the biogenesis of gut granules and the localization of PGP-2, the pathway that directs LMP-1 to gut granules is distinct and more sensitive to alterations in GLO-1 activity.

### GLO-3 and CCZ-1 localize GLO-1

**Fig 8:**
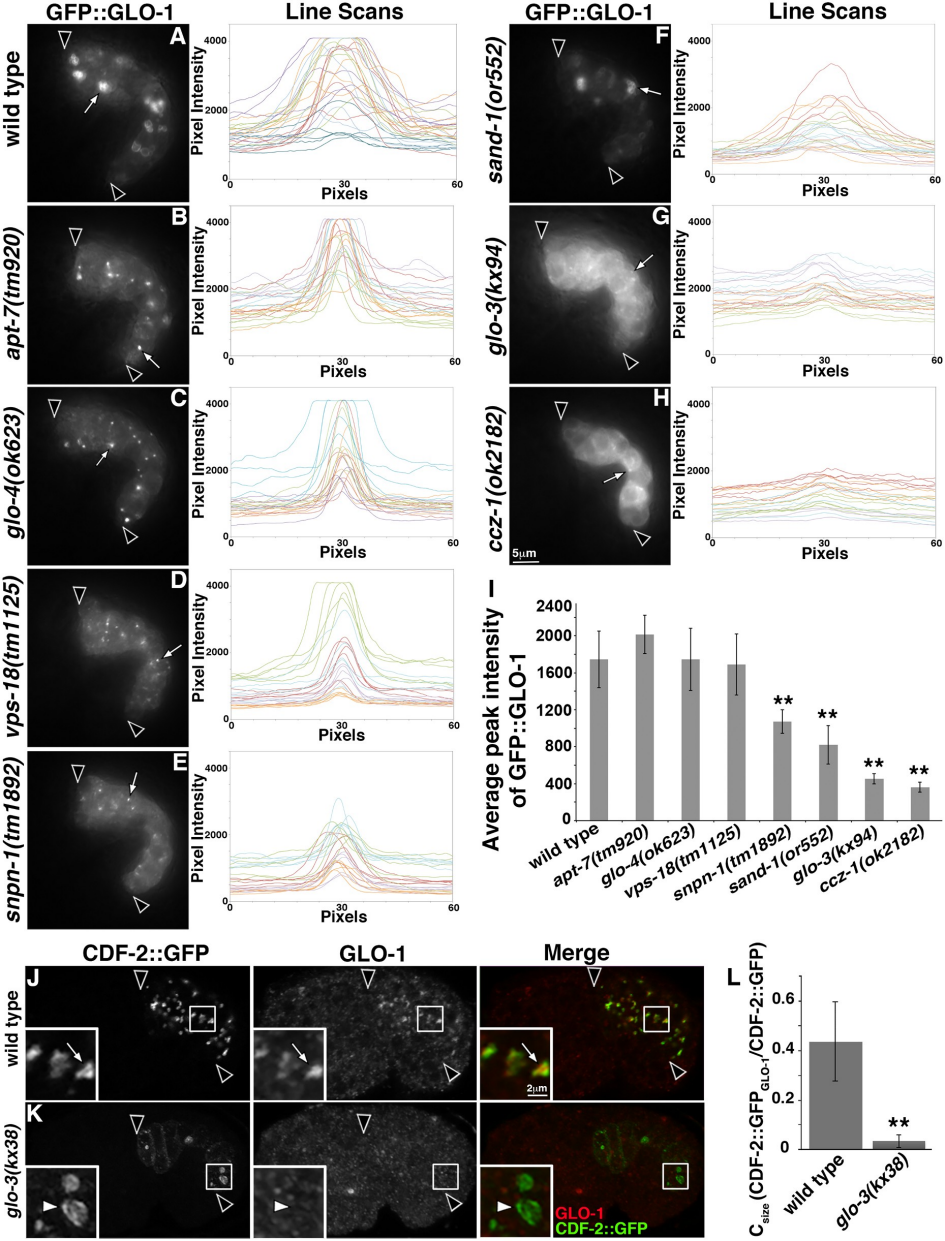
GLO-1 localization is disrupted in *ccz-1(-)* and *glo-3(-)* mutants. (A-H) GLO-1::GFP in living 1.5-fold stage embryos was imaged by wide field microscopy. Embryos were identically imaged and line scan fluorescence intensity centered on 5 puncta (examples are marked with white arrows) from each of 5 different embryos (scans from a single embryo are the same color) was measured. The intestine is flanked by black arrowheads. (I) The background was subtracted from the maximum intensity of each profile in A-H and the mean difference is plotted. Bars are the 95% confidence intervals and ** indicates p≤0.001 by one way ANOVA comparing the mutants to wild type. (J-K) Bean stage embryos expressing CDF-2::GFP were stained with anti-GLO-1 antibodies and imaged with confocal microscopy. (J) CDF-2::GFP marked organelles in wild type contained GLO-1 (white arrows in insets). (K) GLO-1 was lacking from these organelles in *glo-3(kx38)* mutants (white arrowheads in insets). (L) SQUASSH software was used to calculate the area of CDF-2::GFP organelles that contained GLO-1. The mean is plotted and bars are the 95% confidence intervals and ** indicates p≤0.001 by one way ANOVA comparing the *glo-3(-)* mutant to wild type.

Inactive Rabs are GDP-bound and can be extracted from organelle membranes into the cytosol by Rab GDI, while activated, GTP-bound Rabs are resistant to extraction and membrane localized [29, 31]. The restoration of gut granules in *glo-3(-)* and *ccz-1(-)* mutants by GLO-1(D132A) is consistent with GLO-3 and CCZ-1 functioning upstream of GLO-1 activation. We therefore examined whether GFP::GLO-1 was cytoplasmic and diffusely localized or enriched on organelles. For these experiments, we examined living embryos by imaging GFP::GLO-1 and performed line intensity scans to compare the organelle and cytoplasmic signals. In wild type, GFP::GLO-1 was localized to gut granules and relatively little signal was found in the cytoplasm (Fig 8A and 8I). When GFP::GLO-1 was identically imaged, both *glo-3(-)* and *ccz-1(-)* mutants displayed a diffuse signal and GFP::GLO-1 was lacking from prominent puncta (Fig 8G and 8H). Line scans through sites of GFP::GLO-1 enrichment showed that strongly labeled GFP::GLO-1 structures were missing from both mutants (Fig 8G, 8H and 8I). *glo-3(-)* and *ccz-1(-)* mutants lack gut granules (Figs 2, 6 and S2), which could explain the altered distribution of GFP::GLO-1. However, an *apt-7(-)* mutant that disrupts the AP-3 complex [1], a *vps-18(-)* mutant that disrupts the HOPS complex [19], a *snpn-1(-)* mutant that disrupts the BLOC-1 complex [18], and a *glo-4(-)* mutant [1], all lack or have few gut granules and they all displayed a very different GFP::GLO-1 pattern. In these mutants, GFP::GLO-1 was enriched on small puncta and was not diffusely localized (Fig 8B–8E and 8I), indicating that the loss of gut granules per se does not lead to the diffuse localization of GFP::GLO-1 in the *glo-3(-)* and *ccz-1(-)* strains.

CCZ-1 functions with SAND-1(MON1) as a RAB-7 GEF promoting early to late endosome conversion in the conventional lysosomal trafficking pathway [51]. GFP::GLO-1 localization in *sand-1(-)* mutants resembled wild type, albeit with less brightly labeled organelles (Fig 8F and 8I), suggesting that disrupting endosome maturation does not cause the diffuse pattern of GFP::GLO-1 in *glo-3(-)* and *ccz-1(-)* mutants.

The class III *glo-3(kx38)* allele, which generates gut granules marked by PGP-2 (see next section), was used to address whether GLO-1 was localized to gut granules when *glo-3* function was partially, but not completely, disrupted. In fixed wild-type embryos, GFP::GLO-1 was associated with gut granules, whereas it was lacking from PGP-2 marked gut granules in the *glo-3(kx38)* mutant (Fig 7F and S2). To test whether the ectopically expressed and epitope tagged GFP::GLO-1 behaves similar to endogenous GLO-1, we stained wild-type and *glo-3(kx38)* embryos with anti-GLO-1 antibodies. In wild type, GLO-1 localized to gut granules marked by CDF-2::GFP (Fig 8J and 8L). In contrast, GLO-1 was lacking from gut granules in *glo-3(kx38)* mutants (Fig 8K and 8L). Taken together these results show that GLO-3 and CCZ-1 promote the association of GLO-1 with gut granules.

### GLO-1(D132A) localizes to gut granules independently of GLO-3 and CCZ-1

In both wild-type and *glo-1(-)* embryos, GFP::GLO-1(D132A) colocalized with PGP-2 similarly to GFP::GLO-1(+) (Figs 7A, 7B and 7F and S2), demonstrating that the GLO-1(D132A) fast exchange mutant can properly associate with gut granules. Since both *glo-3(-)* and *ccz-1(-)* mutants mislocalized GFP::GLO-1(+) (Fig 7G–7I), we addressed whether GFP::GLO-1(D132A) similarly required *glo-3* and *ccz-1* to localize to gut granules. In the class III *glo-3(kx38)* mutant, GFP::GLO-1(D132A) properly localized to PGP-2 containing compartments at levels similar to when it is expressed in *glo-1(-)* (Figs 7F and S2). In the *glo-3(kx94)* mutant GFP::GLO-1(D132A) often localized to gut granules (Fig 7C and 7F) and GFP::GLO-1(D132A) occasionally associated with gut granules in *ccz-1(-)* mutants, colocalizing with gut granules at lower levels than seen in *glo-1(-)* and the *glo-3(-)* mutants (Fig 7D and 7F). The GLO-3 and CCZ-1 independent localization of GFP::GLO-1(D132A) indicate that these proteins are not absolutely required to localize GLO-1 when its spontaneous exchange activity is increased.

### Gut granules in *glo-3(kx38)*

All of the *glo-1* alleles we have isolated and the *glo-3* class I null alleles, including *glo-3(kx94)*, completely disrupt embryonic gut granule biogenesis (Figs 2 and 6 and Table 1) [1, 44], making it difficult to determine how GLO-1 and GLO-3 function in the pathways generating gut granules. We have isolated a large number of *glo-3(-)* mutants with varying levels of *glo-3(+)* activity and a range of phenotypic effects (Tables 1 and 2) [44]. This allelic series can reveal phenotypes that result from partial *glo-3(+)* activity and point to specific functions for GLO-3 and the GLO-1 Rab it regulates in gut granule biogenesis.

**Fig 9:**
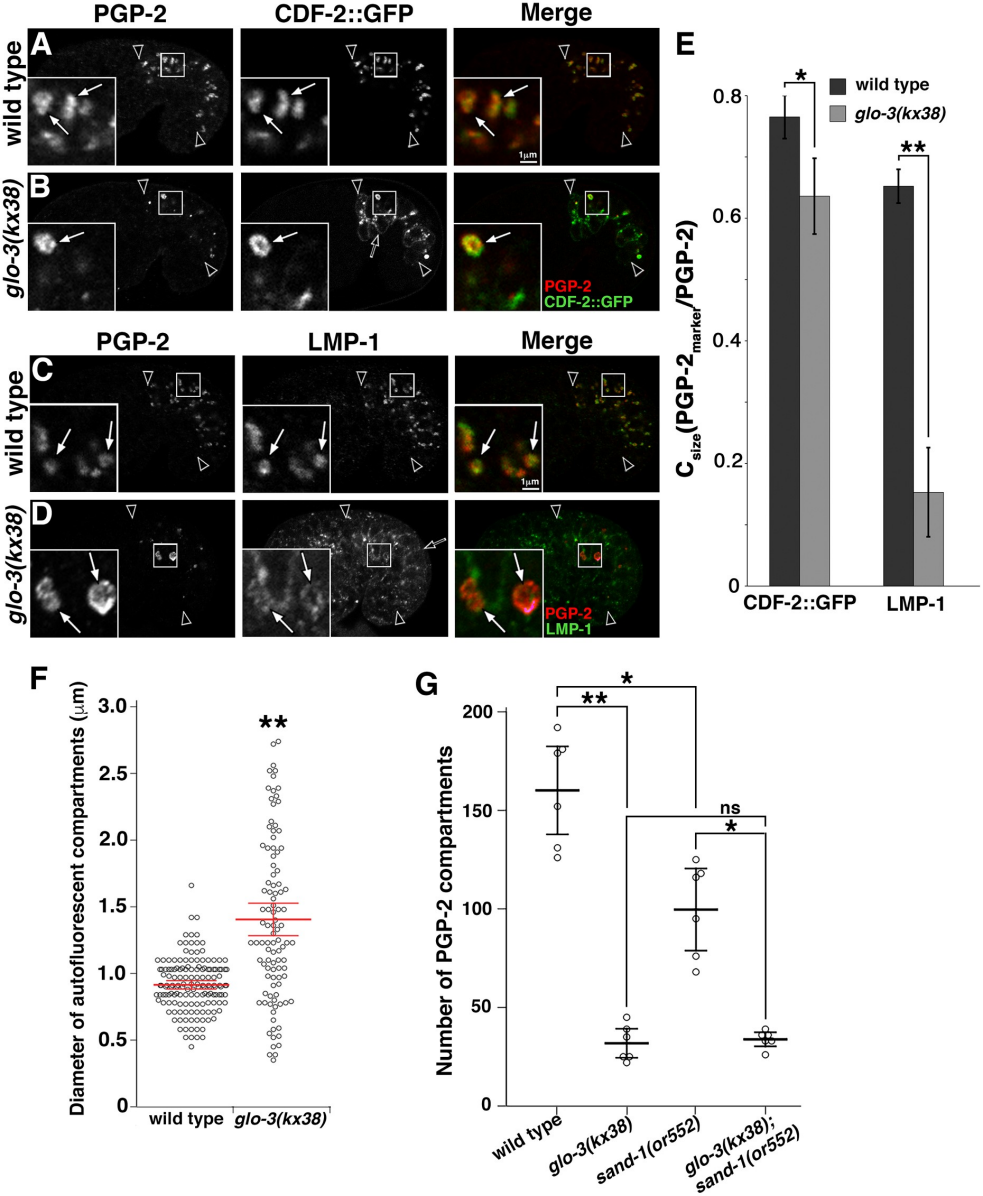
Gut granules in *glo-3(kx38)* mutants. (A-D) Signals from antibodies to PGP-2, RAB-7, LMP-1 and ectopically expressed CDF-2::GFP were acquired in 1.5-fold stage embryos using confocal microscopy. (A-B) In wild type and *glo-3(kx38)* mutants, CDF-2::GFP extensively colocalized with PGP-2 (white arrows in insets), however CDF-2::GFP was also mislocalized to the plasma membrane in *glo-3(kx38)* mutants (black arrow in B). (C-D) While PGP-2 compartments contained LMP-1 in wild type, the anti-LMP-1 signals were weak or lacking in *glo-3(kx38)* mutants (white arrows in insets) and often were located on the plasma membrane (black arrow in D). (A-D) Single optical sections are shown and black arrowheads flank the intestine. (E) SQUASSH software was used to calculate the area of PGP-2 organelles that contained the indicated marker in 5–7 embryos of each genotype. The mean is plotted, bars represent the 95% confidence intervals, * indicates p≤0.05, and ** indicates p≤0.001 by one way ANOVA. (F) Autofluorescent organelles were imaged in living pretzel stage embryos using wide field fluorescence microscopy. The diameters were determined for 25 randomly chosen compartments in six wild-type embryos and all of the compartments (ranging from 9–22 organelles) in six *glo-3(kx38)* mutants. (G) Confocal Z-stacks that span the entire intestine of 1.5-fold stage embryos stained with anti-PGP-2 antibodies were acquired. SQUASSH software was used to quantify the number of PGP-2 compartments in 5 embryos of each genotype. (E-G) In each graph the mean is plotted, bars represent the 95% confidence intervals, * indicates p≤0.05, and ** indicates p≤0.001 by one way ANOVA comparing *glo-3(kx38)* to wild type.

We analyzed the class III allele *glo-3(kx38)* [44], and found that *glo-3(kx38)* mutants generate organelles that have many gut granule characteristics. First, birefringent granules are always generated in *glo-3(kx38)* embryos, albeit at reduced numbers (Table 1). Second, the formation of birefringent granules in *glo-3(kx38)* embryos required the activity of AP-3 (*apt-7*) and BLOC-1 (*glo-2* and *snpn-1*) subunits, as well as other genes (*glo-4* and *pgp-2*) necessary for gut granule biogenesis (Table 3). Third, PGP-2 marked organelles were present in *glo-3(kx38)* mutants and their formation required the same genes (Figs 9B and 9D and S3). Fourth, compartments containing both of the gut granule proteins PGP-2 and CDF-2::GFP were present in *glo-3(kx38)* mutants (Fig 9B and 9E). Finally, the number of organelles marked by PGP-2 was similar to the numbers of birefringent organelles generated in this mutant (Fig 9B and 9G and Table 3).

**Table 3:** Birefringent granule formation in *glo-3(kx38)* double mutants.

Genotype	% of embryos with the specified number of birefringent granules in the intestine				*n*
	0	1–20	21–50	>50	
Wild type	0	0	0	100	41
*glo-3(kx38)*	0	100	0	0	49
*apt-7(tm920)*	38	53	9	0	45
*cup-5(zu223)*	0	0	17	83	42
*glo-2(zu455)*	95	5	0	0	56
*glo-4(ok623)*	100	0	0	0	51
*pgp-2(kx48)*	0	60	31	9	52
*rab-7(ok511)*	0	0	18	82	40
*sand-1(or552)*	0	0	0	100	40
*snpn-1(tm1892)*	100	0	0	0	40
apt-7(tm920); glo-3(kx38)**^,^^##^	76	24	0	0	46
*cup-5(zu223); glo-3(kx38)*	13	87	0	0	40
glo-2(zu455); glo-3(kx38)**	87	13	0	0	40
glo-4(ok623); glo-3(kx38)**	100	0	0	0	42
pgp-2(kx48); glo-3(kx38)**^,^^##^	92	8	0	0	40
*rab-7(ok511); glo-3(kx38)*	0	100	0	0	42
*sand-1(or552); glo-3(kx38)*	0	100	0	0	49
snpn-1(tm1892); glo-3(kx38)**	100	0	0	0	60

Three-fold and later stage embryos were analyzed using polarization microscopy and scored for the presence of birefringent material in the intestine.

** The double mutant displayed significantly less birefringent gut granules than *glo-3(kx38)* (p<0.001, Fisher’s exact test).

^##^ The double mutant displayed significantly less birefringent gut granules than either of the single mutants used to make it (p<0.001, Fisher’s exact test).

We examined whether the gut granule-like organelles in *glo-3(kx38)* had any characteristics of endolysosomes. LMP-1::GFP and two different lysosomal hydrolases, GBA-3::mCherry and CPR-6::mCherry, which is a tagged cathepsin B peptidase [13, 65, 66], were not mislocalized to these compartments (S4 Fig). In addition, birefringent material and CDF-2::GFP remained associated with PGP-2 marked organelles when *glo-3(kx38)* was combined with *cup-5(-)*, which disrupts conventional endolysosomal trafficking and inhibits lysosome formation [55] (S3 Fig and Table 3). Taken together, these observations indicate that gut granules are generated in *glo-3(kx38)* mutants.

While gut granules are present in *glo-3(kx38)* embryos, our work shows that both GFP::GLO-1 and endogenous GLO-1 are lost from these compartments (Figs 7F and 8K and S2). Therefore an analysis of this mutant can reveal effects on LRO biogenesis when *glo-3(+)* activity is reduced and GLO-1 is lacking from gut granules. The most obvious effect of *glo-3(kx38)* is on gut granule number and size; compared to wild type, the number of gut granules marked by PGP-2 was reduced by more than 80% and the average gut granule diameter was 55% larger (Fig 9F and 9G). To determine if *glo-3(kx38)* disrupts protein trafficking, we analyzed the localization of gut granule markers in *glo-3(kx38)* mutants. LMP-1 is localized to both gut granules and conventional lysosomes [13], and in *glo-3(kx38)* mutant embryos LMP-1 was mislocalized to the plasma membrane and lacking or only weakly associated with gut granules (Fig 9C–9E). CDF-2::GFP remained associated with gut granules in *glo-3(kx38)* mutants (Fig 9B and 9E). However, CDF-2::GFP was mislocalized to the plasma membrane and what are likely conventional lysosomes based upon their morphology, location, and enlargement in *cup-5(-)* mutants (Figs 9B and S3). These analyses show that the localization of CDF-2::GFP and LMP-1 are sensitive to reduction in *glo-3* activity, while PGP-2 appears to be unaffected. Notably, the presence of gut granules in *glo-3(kx38)* mutants indicates that the enrichment of GLO-1 on gut granules is not necessary for their biogenesis.

### RAB-7 associates with gut granules in *glo-3(kx38)*

**Fig 10:**
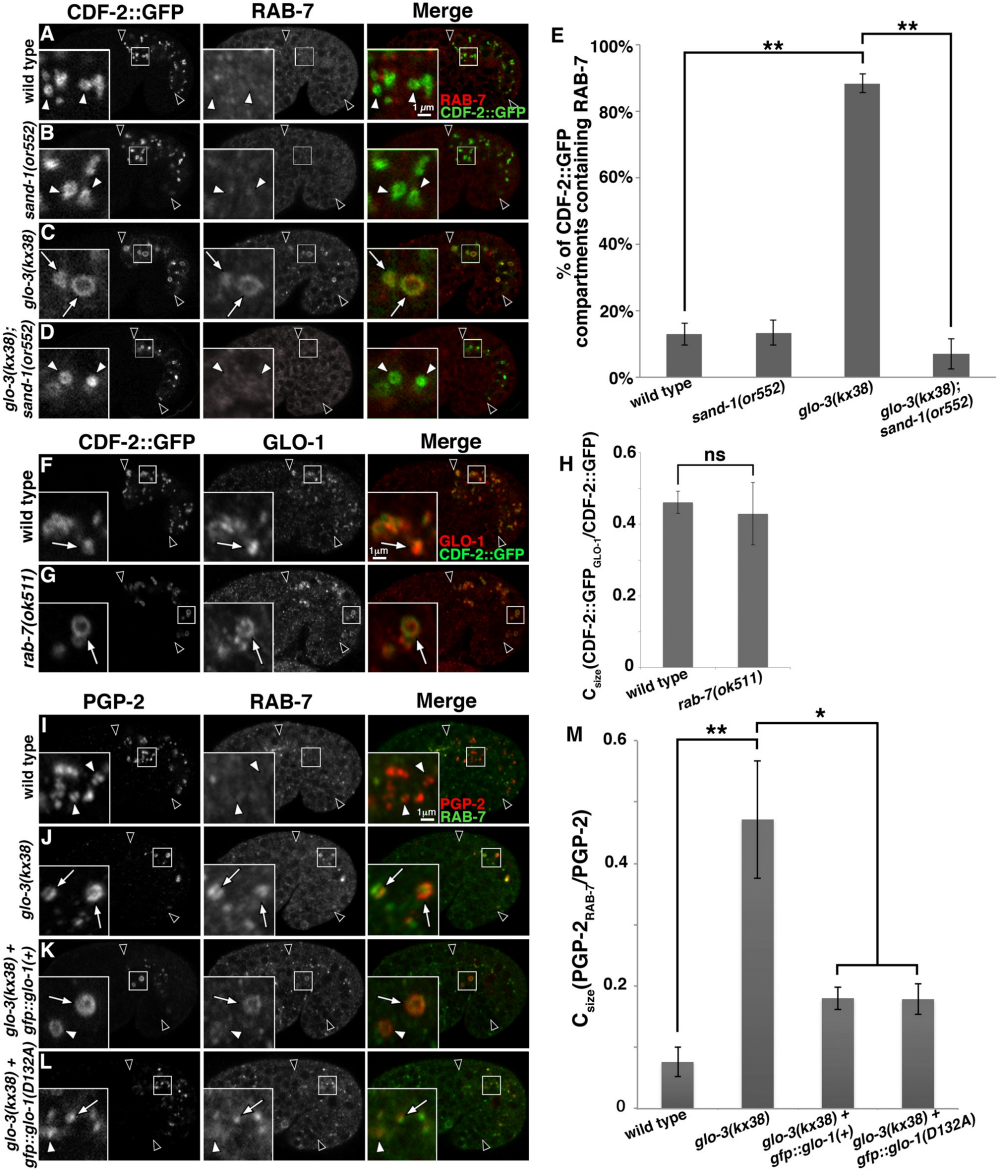
RAB-7 associates with gut granules in *glo-3(kx38)* mutants. (A-D) Signals from antibodies to RAB-7 and ectopically expressed CDF-2::GFP were acquired in 1.5-fold stage embryos using confocal microscopy. RAB-7 was not enriched on CDF-2::GFP marked gut granules in wild type or *sand-1(or552)* 1.5-fold stage embryos (white arrow heads in insets). The RAB-7 that associated with gut granules in *glo-3(kx38)* mutants (white arrows in insets) was lacking in *glo-3(kx38); sand-1(or552)* double mutants. (E) Colocalization was scored by randomly selecting at least 10 CDF-2::GFP organelles and assessing the presence of the RAB-7 signal in each of 10–12 embryos of each genotype. In *glo-3(kx38)* mutants CDF-2::GFP associated with both gut granules and lysosomes (S3B Fig). Only gut granules, which have a diameter larger than 0.55μm, were scored. The mean is plotted and bars represent the 95% confidence intervals and ** indicates p≤0.001 by one way ANOVA. (F-G) 1.5-fold stage embryos expressing CDF-2::GFP were stained with anti-GLO-1 antibodies and imaged with confocal microscopy. GLO-1 was similarly associated with gut granules in wild type and *rab-7(-)* mutants (white arrows in insets). (H) SQUASSH software was used to calculate the area of CDF-2::GFP organelles that contained GLO-1 in 5 embryos of each genotype. The mean is plotted, bars represent the 95% confidence intervals, ns indicates p>0.05 by one way ANOVA. (I-L) Signals from antibodies to PGP-2 and RAB-7 were acquired in 1.5-fold stage embryos using confocal microscopy. The expression of GFP::GLO-1(+) or GFP::GLO-1(D132A) led to the diminished localization of RAB-7 on PGP-2 compartments in *glo-3(kx38)* mutants. (M) SQUASSH software was used to calculate the area of PGP-2 organelles that contained RAB-7 in 5–7 embryos of each genotype. The mean is plotted, bars represent the 95% confidence intervals, * indicates p≤0.05 and ** indicates p≤0.001 by one way ANOVA. In all images single optical sections are shown and black arrowheads flank the intestine.

Due to the ability of small GTPases that direct intracellular trafficking to regulate each other’s localization [31, 67, 68], we investigated whether the reduction in *glo-3* activity and loss of GLO-1 from gut granules in *glo-3(kx38)* mutants had any effects on the gut granule localization of other Rab and Arf GTPases. The early endosomal GFP::RAB-5, apical recycling endosomal GFP::RAB-11.1, basolateral recycling endosomal GFP::RAB-10, and lysosomal ARL-8::GFP were not mislocalized to gut granules in *glo-3(kx38)* mutants (S4 Fig). In contrast, a significant fraction of gut granules in *glo-3(kx38)*, but not wild-type embryos, accumulated the late endosomal GFP::RAB-7 (S4 Fig). Confirming the result with the ectopically expressed and tagged protein, endogenous RAB-7 similarly mislocalized to gut granules in *glo-3(kx38)* mutants (Fig 10C, 10E, 10J and 10M). Gut granules in class IV *glo-3(-)* mutants accumulated RAB-7 as well (S5 Fig). The activation and localization of RAB-7 to endosomes is mediated by CCZ-1/SAND-1(MON1) [42, 51, 56]. RAB-7 was lacking from *sand-1(-); glo-3(kx38)* gut granules (Fig 10D and 10E), consistent with SAND-1 promoting the association of RAB-7 with gut granules in *glo-3(kx38)* mutants.

We addressed whether RAB-7 has a role in the formation of gut granules in *glo-3(kx38)* mutants. Suggesting that it does not, we found that the formation of birefringent gut granules was not disrupted in *glo-3(kx38); rab-7(ok 511)* double mutants (Table 3). *sand-1(-)* mutations disrupt RAB-7 activity and localization [42], and when combined with *glo-3(kx38)* did not alter the formation of birefringent gut granules (Table 3), the number of PGP-2 marked gut granules (Fig 9G), or the colocalization of the gut granule proteins PGP-2 and CDF-2::GFP (S3 Fig). Together, these results strongly suggest that the presence of RAB-7 on gut granules in *glo-3(kx38)* does not substantially impact their biogenesis.

*rab-7(-)* mutants generate gut granules whose morphology, number, and protein composition resemble the gut granules in *glo-3(kx38)* mutants [19], suggesting that RAB-7 might play a role in recruiting GLO-1. However, we found that GLO-1 was present on gut granules in *rab-7(ok511)* mutants (Fig 10F–10H), indicating RAB-7 is not necessary for the recruitment of GLO-1 to gut granules.

To further investigate the functional relationships between GLO-1 and RAB-7 we ectopically expressed GFP::GLO-1(+) and GFP::GLO-1(D132A) in *glo-3(kx38)* mutants and found that both led to a significant decrease in the association of RAB-7 with gut granules (Fig 10I–10M). These results point to a role for GLO-1 in preventing the association of RAB-7 with gut granules and suggest that the association of RAB-7 with gut granules in *glo-3(kx38)* mutants could result from the loss of GLO-1 from these organelles. One mechanism by which GLO-1/GLO-3 could restrict RAB-7 from gut granules would be through the recruitment and/or activation of the RAB-7 GTPase activating protein (GAP). Currently, the RAB-7 GAP is not known in *C*. *elegans*, however the RAB-5 GAP TBC-2 has RAB-7 GAP activity *in vitro*, and genetic studies are consistent with it functioning as a RAB-7 GAP [69–71]. However, *tbc-2(-)* mutants did not lead to the mislocalization of RAB-7 to gut granules or obvious defects in gut granule protein trafficking (S6 Fig).

## Discussion

GLO-1 and related Rab32/38 proteins were initially identified due to their role in the biogenesis of LROs in mammals [24, 27], *Drosophila melanogaster* [25], and *C*. *elegans* [1]. More recently Rab32 family members have been implicated in autophagy [72, 73], phagocytosis of bacterial pathogens [74–77], and endocytosis and proteolytic degradation [78]. In *C*. *elegans*, *glo-1(-)* early embryos are defective in the autophagic degradation of paternal mitochondria [79–81]. In the nervous system, *glo-1(-)* adults show decreased numbers of RAB-7 labeled compartments [82], altered necrosis [83], and defects in synapse formation and neuronal morphology [84]. All of these more recently identified roles for Rab32 family members, including GLO-1, could result from functions in the conventional endolysosomal pathway. In fact, many of the factors originally characterized as having a role in LRO biogenesis are now known to support conventional endolysosomal trafficking [1, 19, 85–89]. Notably, we did not detect a significant role for GLO-1 in the transport of cargo through conventional endolysosomes and instead show that GLO-1 functions to direct protein cargo away from this pathway toward gut granules (Figs 2 and 3). Our data support an LRO restricted role for GLO-1 in intestinal cells and we suggest that processes mediated by gut granules impact developmental and physiological processes outside the intestine or that other *C*. *elegans* cell types possess LROs, with different functions than gut granules, whose formation requires GLO-1.

Similar to other Rabs [29, 31], we show that the GTP bound form of GLO-1 is active in gut granule formation (Fig 5). Following GTP hydrolysis, most Rabs will remain in the inactive form due to their low intrinsic rate of exchange of GDP for GTP [90]. In mammals, Rab32/38 guanine nucleotide exchange is catalyzed by BLOC-3, a heterodimeric complex composed of HPS1 and HPS4 [33]. BLOC-3 subunits show sequence and functional homology with CCZ-1/SAND-1(MON1), which function as a heterodimeric GEF for RAB-7 [33, 38, 39, 41, 42, 91, 92]. In addition to interacting with SAND-1(MON1), we find that CCZ-1 can directly bind to GLO-3 (Fig 1). We have previously shown that CCZ-1, but not SAND-1 or RAB-7, is required for gut granule biogenesis, and that a point mutation in GLO-1 predicted to increase the rate of spontaneous guanine nucleotide exchange restores of autofluorescent organelles in *ccz-1(-)* and *glo-3(-)* mutant adults [19]. Here we definitively show that the function of CCZ-1 and GLO-3, but not other gut granule biogenesis factors, is bypassed by the GLO-1 fast-exchange mutant (Figs 6 and 7 and Tables 1 and 2). Furthermore, GFP::GLO-1 is diffusely localized in *ccz-1(-)* and *glo-3(-)* mutants and GLO-3 functions in the recruitment to and/or stabilization of GLO-1 on gut granules (Fig 8). Loss of GLO-1 GEF activity should result in the accumulation of GLO-1 in the GDP bound form, which would be extracted from organelle membranes into the cytosol by Rab GDI [29, 31]. Together, these results strongly suggest a CCZ-1 and GLO-3 function as a GEF that activates and localizes GLO-1.

While the GLO-1(D132A) fast-exchange mutant promoted the proper localization of some gut granule proteins in *glo-3(-)* and *ccz-1(-)* mutants, the localization of GLO-1(D132A) was reduced and LMP-1 was noticeably absent from these organelles (Fig 7). Rab GEFs are known to play important roles localizing their Rab substrates, and it is currently unknown whether this is purely through catalyzing nucleotide exchange or through other functions such as physical interactions that recruit the Rab [29, 31]. Our observations suggest the latter possibility for GLO-3 and CCZ-1. It is also possible that the higher level of GLO-1(D132A) gut granule association when GLO-3 and CCZ-1 are present, could result from these factors promoting the GTP, membrane localized form of the fast exchange mutant. LMP-1 trafficking to gut granules requires the function of the AP-3 adaptor complex, while other gut granule proteins can localize to gut granules independently of AP-3 [13]. The defects in LMP-1 localization could result from GLO-3 and CCZ-1 functioning in the AP-3 pathway independently of regulating GLO-1. The Rab GEFs, Rabin8, VARP, and possibly the TRAPP complexes, have GEF-independent roles in membrane dynamics [93–95]. Additionally, the activation cycle of GLO-1(D132A) might not fully restore wild-type GLO-1 activity, disrupting the delivery of LMP-1 to gut granules. In support of this possibility, biochemical and genetic analysis of analogous fast-exchange mutations in RAB-7 show that they cause decreased RAB-7 function [61, 96].

Our *in vivo*, genetic studies suggest that the GEF activity of CCZ-1 for two different Rabs is controlled by its interaction partner. CCZ-1 and SAND-1(MON1) have amino-terminal longin domains that mediate heterodimerization and nucleotide exchange by RAB-7 [40]. Our *in vitro* binding studies show that the amino terminal region of CCZ-1 containing the longin domain is sufficient to interact with GLO-3 (Fig 1). This suggests mutually exclusive binding of GLO-3 and SAND-1(MON1) to CCZ-1 due to competition for the same binding site on CCZ-1, which could regulate Rab substrate specificity. GLO-3 has an amino-terminal longin-like domain [19], however in our assays this region does not show strong interactions with CCZ-1 (Fig 1). Similar to CCZ-1/SAND-1(MON1), the TRAPPII and TRAPPIII complexes are Rab GEFs that are composed of longin domain containing subunits [97, 98]. Interestingly, it has recently been shown that substitution of longin domain containing subunits can alter the Rab GEF specificity of the TRAPP complexes [99–103]. In the CCZ-1/SAND-1(MON1) complex, SAND-1(MON1) makes the majority of contacts with RAB-7 [40]. Thus, substitution of GLO-3 for SAND-1(MON1) in a heterodimer with CCZ-1 could redirect the specificity of a CCZ-1 containing GEF from RAB-7 to GLO-1. In support of this idea *ccz-1(-)* mutants show defects in gut granule protein localization not seen in *sand-1(-)*, *glo-1(-)*, or *glo-3(-)* single mutants (Fig 4). However, *ccz-1(-)* is phenocopied by *sand-1(-); glo-3(-)* and *sand-1(-); glo-1(-)* double mutants (Fig 4). The similar effects of *glo-1(-)*, *glo-3(-)*, and *ccz-1(-)* mutants on gut granule protein localization (Fig 2) [19], the cytoplasmic mislocalization of GLO-1 in *glo-3(-)* and *ccz-1(-)* mutants (Fig 8), the ability of the fast exchange GLO-1(D132A) mutants to restore gut granules in *glo-3(-)* and *ccz-1(-)* mutants (Figs 6 and 7 and Tables 1 & 2), and the ability of GLO-3 to directly bind CCZ-1 (Fig 1), strongly support the model that CCZ-1, by functioning with GLO-3, can regulate GLO-1.

In mammalian cells the Ccz1/Mon1(SAND-1) complex acts as a GEF for Rab7 and the HPS1/HPS4(BLOC-3) complex acts as a GEF for the GLO-1 homologues Rab32 and Rab38 [33, 43]. *C*. *elegans* does not appear to similarly segregate Rab7 and Rab32/38 GEF activities. However, while mammalian Rab32/38 expression is restricted to a subset of cell types [1, 104–107], BLOC-3 subunits are ubiquitously expressed [34–36], and BLOC-3 mutants disrupt normal endolysosome distribution [108]. This supports the possibility that mammalian HPS1 and HPS4 regulate Rabs promoting both LRO and conventional endolysosome biogenesis, similarly to CCZ-1 in *C*. *elegans*.

*glo-3(-)* weak mutants show SAND-1 dependent mislocalization of RAB-7 to gut granules (Figs 10 and S4 and S5). If GLO-3 and SAND-1 compete for CCZ-1 binding then the mislocalization of RAB-7 could result from excessive CCZ-1/SAND-1(MON1) heterodimer formation and RAB-7 activation when GLO-3 levels are reduced. Alternatively, GLO-3 might inhibit RAB-7 association with gut granules by recruiting or activating a negative regulator of RAB-7 activity. Ectopically expressed GLO-1 reduced the association of RAB-7 with gut granules in *glo-3(-)* mutants (Fig 10), suggesting the possibility of a Rab cascade in gut granule biogenesis [30, 31]. We found that *rab-7(-)* mutants, despite generating gut granules that phenotypically resemble gut granules in weak *glo-3(-)* mutants (Fig 9) [19], properly localize GLO-1 to gut granules (Fig 10). Disrupting the activity of *tbc-2*, which encodes a possible RAB-7 GAP [69–71], did not lead to the gut granule association of RAB-7. However, it is possible that RAB-7 acts in concert with other factors to recruit the GLO-1 GEF and that activated GLO-1 recruits a different RAB-7 GAP to mediate an exchange of RAB-7 on late endosomes for GLO-1 on gut granules.

Rab GEFs are currently thought to be the major factors determining the subcellular localization of Rabs [33, 44, 109–112]. GLO-3 associates with gut granules [44], putting it in the correct position to direct GLO-1 localization. However, if GLO-3 and CCZ-1 function as a GLO-1 GEF, how does GLO-1(D132A) localize to gut granules in the absence of these proteins? Analogous fast exchange Rab7 and RAB-2 mutants are properly localized when the activity of their respective GEFs is lacking [113, 114], suggesting that GEFs are not essential for the localization of Rabs that have an increased rate of nucleotide exchange. We know little about the identity and function of factors other than GEFs that impact the recruitment and/or stabilization of most Rabs, but they have been suggested to include Rab-GDI displacement factors or Rab effectors [115–118].

It is likely that each Rab utilizes a distinct set of interacting factors and mechanisms to ensure its correct spatiotemporal distribution [29, 31]. Rab GEFs are typically not membrane anchored, a key characteristic of a membrane targeting receptor. Possibly there are integral membrane proteins that function as Rab receptors or modify the organelle membrane to promote Rab localization. The identification and characterization of these factors will be critical for understanding how organelles acquire their functional identity.

## Materials and methods

### Nematode strains and culture

*C*. *elegans* strains were cultured at 22°C on NGM media seeded with *E*. *coli* strain OP50 [119]. N2 was used as the wild type and all mutant alleles were generated in this strain. The following mutations were used: *apt-7(tm920)*, *ccz-1(ok2182)*, *cup-5(zu223)*, *glo-1(zu437)*, *glo-2(tm592)*, *glo-3(gk582755)*, *glo-3(kx38)*, *glo-3(kx94)*, *glo-3(syb272)*, *glo-3(zu446)*, *glo-4(ok623)*, *rab-7(ok511)*, *sand-1(or552)*, *snpn-1(tm1892)*, *tbc-2(tm2241)*, *unc-36(e251)*, *vps-18(tm1125)*. Wormbase (www.wormbase.org) hosts descriptions of each allele. The following transgenes were used: *amIs4[cdf-2p*::*cdf-2*::*gfp; unc-119(+)]* [46], *cbgIs98[pept-1p*::*gfp*::*rab-11*.*1; unc-119(+)]* [120], *kxEx9[glo-1p*::*gfp*::*glo-1; Rol-6*^*D*^*]* [1], *kxEx141[cpr-6p*::*cpr-6*::*mCherry; Rol-6*^*D*^*]* [13], *kxEx148[gba-3p*::*gba-3*::*mCherry; Rol-6*^*D*^*]* [13], *kxEx223[glo-1p*::*gfp*::*glo-1(T25N); Rol-6*^*D*^*]* (this work), *kxEx230[glo-1p*::*gfp*::*glo-1(Q71L); Rol-6*^*D*^*]* (this work), *kxEx252[vha-6p*::*gfp*::*glo-1(D132A); Rol-6*^*D*^*]* [19], *kxEx254[vha-6p*::*gfp*::*glo-1(I133F); Rol-6*^*D*^*]* [19], *kxEx272[glo-1p*::*gfp*::*glo-1(D132A); Rol-6*^*D*^*]* (this work), *kxEx273[glo-1p*::*gfp*::*glo-1(I133F); Rol-6*^*D*^*]* (this work), *pwIs50[lmp-1p*::*lmp-1*::*gfp; unc-119(+)]* [55], *pwIs72[vha-6p*::*gfp*::*rab-5; unc-119(+)]* [1], *pwIs170[vha-6p*::*gfp*::*rab-7; unc-119(+)]* [121], *pwIs206[vha-6p*::*gfp*::*rab-10; unc-119(+)]* [121], *pwIs503[vha-6p*::*mans*::*gfp; unc-119(+)]* [121], *tdEx2[arl-8p*::*arl-8*::*gfp; Rol-6*^*D*^*]* [52].

### Genetic manipulations

Integrated (*Is*) and extrachromosomal (*Ex*) transgenes, present in otherwise wild-type strains, were moved into mutant backgrounds by crossing hermaphrodites containing the transgenes with males homozygous or heterozygous for the mutation. The presence of the mutation in the resulting strain was confirmed by the presence of the mutant phenotype, or in cases where this was modified by the transgene, by PCR and/or DNA sequencing. To generate double mutants containing *glo-3(kx38)*, transheterozygous individuals were allowed to self fertilize and progeny that were homozygous for *glo-3(kx38)*, as evidenced by the number of birefringent gut granules, and heterozygous for the other mutation, were isolated. The homozygous double mutants that exhibited the other mutant phenotype were then isolated from these strains. In cases where one Glo phenotype masked another, we confirmed the presence of the masked mutation using PCR/DNA sequencing. In all cases, single and double mutants were homozygous for each mutation except strains containing *rab-7(ok511)*, *cup-5(zu223)*, and some strains containing *ccz-1(ok2182)*, which were kept heterozygous due to the recessive maternal effect lethality or severe growth defect caused by these mutations [54, 57, 86]. In cases where strains heterozygous for these mutations were used, we identified homozygous mutant adults by the presence of large DIC refractile granules within embryos in their uterus or a linked recessive marker. Mutant embryos produced by homozygous *rab-7(-)*, *cup-5(-)*, and *ccz-1(-)* parents display these morphologically distinct structures [54, 57, 86]. *unc-36(e251)* was linked to *cup-5(-)* and we found that it did not alter gut granule biogenesis in any of our assays. *glo-3(gk582755)* was identified in an ongoing screen of Million Mutation strains for defects in gut granule number and/or morphology. The Glo phenotype of strain VC40338 mapped to the X chromosome and did not complement the Glo phenotypes of *glo-3(zu446)*. The *glo-3(gk582755)* mutation causes a GLO-3(N279K) substitution and was backcrossed 3 times to N2 before being characterized. CRISPR-Cas9 gene editing was carried out by SunyBiotech (Fuzhough City, Fujian, China) to generate *glo-3(syb272)*, which precisely removes the entire *glo-3* coding sequence. Sanger sequencing verifying the presence of the deletion was carried out by Genewiz (South Plainfield, NJ, USA). The resulting sequence TTCgAGGTAAACTCGTTCAAA—ATAATTTATATTTACAAGTAT flanked the deletion (marked by—). The g denotes a mutation created to destroy the PAM site. *syb272* was backcrossed 4 times to N2 before being characterized. To knock down the expression of *rab-7* we used RNAi feeding protocol 1 described in [122] and clones from the Ahringer RNAi library (Source Bioscience, Nottingham, UK). The effects of *rab-7* RNAi were not seen in embryos treated with F33E2.4(RNAi), which targets a gene not required for gut granule biogenesis. In RNAi experiments, inhibition of *rab-7* activity was confirmed by the presence of DIC refractile granules.

### Microscopy

Widefield polarization and fluorescence microscopy was carried out with a Zeiss AxioImager.M2 and images were captured with an AxioCam MRm digital camera controlled by AxioVision 4.8 software (Zeiss, Thornwood, NY). Confocal fluorescence microscopy was carried out with a Zeiss LSM710 laser scanning confocal microscope. Embryos were imaged with 100X or 63X Plan-Apochromat 1.4 NA objectives and adults were imaged with a 40X Plan-Apochromat 1.3 NA objective.

Adults were mounted on 3% agarose pads and immobilized with 10mM levamisole (Sigma Aldrich, St. Louis, MO). Autofluorescent gut granules were imaged with a Zeiss 38 filter (GFP, excitation, BP 470/40; emission, BP 525/50), a Zeiss 45 filter (mCherry, excitation, BP 560/40; emission, BP 630/75), or a 488 laser line. Z-stacks of the intestine were captured and maximum intensity projections of ½ or the entire depth of the intestine are shown. In adults expressing GFP tagged proteins, the Zeiss 45 filter was used to visualize gut granules.

Living embryos were mounted in H_2_O on 3% agarose pads. Body movements in embryos do not begin until after the 1.5-fold stage. To acquire images of late stage embryos, excess respiring OP50 bacteria was added to induce hypoxia and immobilization. Birefringent material was visualized with polarization optics. Maximum intensity projections of Z-stacks capturing all of the birefringent material within the intestine are shown. GFP and mCherry markers were imaged by confocal microscopy in living 1.5-fold stage embryos using the 488 and 561 laser lines. GFP, mCherry, and autofluorescence in living embryos were imaged using widefield microscopy with Zeiss, 38, Zeiss 45 and Zeiss 49 (DAPI, excitation, G 365; emission, 445/50) fluorescence filters, respectively.

To characterize the pattern of GFP::GLO-1 in living embryos, GFP::GLO-1 signals in each strain were captured using widefield fluorescence microscopy using identical exposure settings. Z-stacks through the top half of the intestine were captured. The Fiji software plot profile tool centered on randomly selected puncta was used to generate intensity profile histograms [123]. The intensity value for each punctum was calculated by subtracting the average of the 10 lowest intensity values from the peak value in each 60 pixel intensity histogram. Widefield fluorescence Z-stacks of autofluorescent gut granules in pretzel stage embryos were imaged with a Zeiss 49 filter. The diameter of these organelles was determined using Zeiss AxioVision software.

Embryos were fixed in -20°C MeOH following a freeze-crack as described [124]. The intrinsic fluorescence of GFP was used to visualize the distribution of GFP tagged proteins after fixation. Antibodies to GLO-1 [1], LMP-1 [125], PGP-2 [47], RAB-5 [126], and RAB-7 [127] were used. Z-stacks through the intestine of fixed LMP-1::GFP expressing embryos were imaged using widefield microcopy with a Zeiss 38 filter. Using the plasma membrane localization of LMP-1::GFP to identify individual cells, we manually quantified the number of lysosomes located within the 4 cells that make up Int 2 and 3. To simultaneously image the localization of CDF-2::GFP, PGP-2, and LMP-1 in embryos, we used secondary antibodies marked with DyLight 405 and Rhodamine Red (Jackson ImmunoResearch, West Grove, PA). These were imaged with confocal microscopy using the 405, 488, and 561 laser lines. Widefield fluorescence microscopy was used in some experiments to capture GFP, Alexa 488, or Rhodamine Red fluorescence with Zeiss 45 or Zeiss 49 filters. The number of PGP-2 marked organelles in individual embryos was quantified using SQUASSH software analysis of confocal Z-stacks spanning the entire intestine [64]. Using confocal Z-stacks, the diameter of CDF-2::GFP or anti-LMP-1 marked organelles was determined using Zeiss Zen Blue 2012 software. In *glo-3(kx38)* containing strains, only the diameter of CDF-2::GFP organelles that also contained PGP-2 were measured, as CDF-2::GFP was mislocalized to non-PGP-2 containing lysosomes in *glo-3(kx38)* mutants. To determine endolysosome size, only LMP-1 containing organelles that lacked the gut granule marker PGP-2 were measured.

For colocalization studies, two or three channel confocal Z-stacks were acquired and analyzed. As noted in the figure legends, either manual or automated colocalization scoring was performed. In some experiments, randomly selected organelles labeled by one marker were manually scored for the presence of a second marker. Individual organelle signals were scored as colocalizing if they overlapped by more than 50%. The colocalization per embryo was calculated and these values were used to determine the mean colocalization shown in the graphs. In other experiments, SQUASSH software was used to segment the Z-stack and every identified organelle was used in the analysis (typically 120–160 gut granules per wild-type embryo). The resulting C_size_ measurement of colocalization represents the fraction of the total volume of one marker that overlapped with the second marker [64]. For example, C_size_(PGP-2_LMP-1_/PGP-2) refers to the area of PGP-2 that overlapped with LMP-1 divided by the total area of PGP-2, and represents the proportion of PGP-2 that colocalizes with LMP-1. The C_size_ per embryo was calculated and these values were used to determine the mean colocalization shown in the graphs.

One way ANOVAs were carried out in Microsoft Excel for Mac 2011. Bonferroni or Tukey-Kramer post hoc tests were used when making 3 or more comparisons. Bar graphs were generated with Excel for Mac 2011 and dot plots were made with R (version 3.1.2) Beeswarm (Version 0.1.6). Figures were constructed with Photoshop CS2 and representative images used to determine marker colocalization and organelle presence, number, or size is shown. Brightness and contrast adjustments were uniformly applied to each panel.

### Yeast 2-hybrid assays

The *S*. *cerevisiae* EGY48 strain was used for all 2-hydrid assays [128]. The DupLEX-A yeast 2-hybrid system was used according to the manufacturer’s instructions (Origene Technologies, Rockville, MD, USA). The bait plasmids pEG202 and pEG202-NLS encoding LexA DNA binding domain fusions and prey plasmid pJG4-5 encoding B42 transcription activation domain fusions were used. A full-length *glo-3* cDNA was PCR amplified from pDONR/Zeo-*glo-3* using (*italics* are homologous to vector sequences and **bold** hybridize with the coding sequence) P1129 5’*CAGATTATGCCTCTCCCG*CC**ATGTTTGGTTATGTTGTTGTTAATGAAC**3’ and P1130 5’*GCGAAGAAGTCCAAAGCTTC*GG**TTATTTTAACTGTTTTAACACGCATTCC**3’ with Q5 High Fidelity DNA polymerase (NEB, Ipswich, MA, USA) and inserted into pJG4-5 digested with EcoRI and XhoI using NEBuilder HiFi DNA Assembly Cloning as described by the manufacturer (NEB). A full-length *ccz-1* cDNA was amplified from pDONR/Zeo-*ccz-1* using P1119 ‘*AACGGCGACTGGCTGG*CC**ATGGAGTCGATTGCAAATCCATTG**3’ and P1120 5’*CTTGGCTGCAGGTCGAC*GG**TCAACTAAAAAATATGGCTTCGAAATGGG**3’ with Q5 High Fidelity DNA polymerase (NEB) and inserted into pEG202 digested with EcoRI and XhoI, using NEBuilder HiFi DNA Assembly Cloning as described by the manufacturer (NEB). Sequencing of the resulting plasmids showed that the coding sequences lacked mutations and were in-frame with the DNA binding or transcription activation domains (Genewiz, South Plainfield, NJ, USA). Lithium acetate mediated transformation was used to simultaneously introduce combinations of plasmids into EGY48. LEU2 reporter expression was assessed by growing strains in 2% dextrose lacking histidine, tryptophan, and uracil liquid media overnight, diluting to 1.0 OD600, and spotting this and serial dilutions on 2% dextrose or 2% galactose/1% raffinose plates lacking leucine, histidine, tryptophan, and uracil at 30°C for 3 days. The pSH18-34 reporter plasmid was used to assess lacZ expression by growing strains on 2% dextrose or 2% galactose/1% raffinose plates containing 80μg/ml X-Gal and lacking histidine, tryptophan, and uracil at 30°C for 3 days.

### Recombinant proteins and GST pull downs

Full length (1–1821 bp) and amino terminal encoding (1–657 bp) *glo-3* cDNAs were inserted into pGEX4T1 with BamHI and XhoI. Full length (1–1584 bp) and amino terminal encoding (1–600 bp) *ccz-1* cDNAs were inserted into pET28a with BamHI and XhoI. Recombinant GST-GLO-3 proteins were expressed in Rosetta(DE3) bacterial cells and purified with glutathione-Sepharose beads (GE Healthcare Bio-Sciences Pittsburg, PA) according to the instructions provided by the supplier. Recombinant His6-CCZ-1 proteins were expressed in Rosetta(DE3) bacterial cells and purified with Chelating Sepharose Fast Flow (GE Healthcare Bio-Sciences Pittsburg, PA) according to the instructions provided by the supplier. Purified GST or GST-GLO-3 proteins (2.5 μg of each) were immobilized on glutathione-Sepharose beads and then pre-incubated with blocking buffer (5% BSA, 100mM Tris-HCl PH7.5, 150mM NaCl, 10mM DTT, 0.05% NP40, 1mM PMSF) at 4°C for 1h, then incubated with His6-CCZ-1 proteins in the binding buffer (1%BSA, 100mM Tris-HCl PH7.5, 150mM NaCl, 10mM DTT, 0.05% NP40, 1mM PMSF) at 4°C for 4 h. After extensively washing with washing buffer (100mM Tris-HCl PH7.5, 150mM NaCl, 10mM DTT, 0.05% NP40, 1mM PMSF), bound proteins were resolved on sodium dodecyl sulphate (SDS) polyacrylamide gels (SDS-PAGE) and visualized by Western Blot.

### GLO-1 point mutations

Mutations predicted to activate (Q71L) or inactivate (T25N) GLO-1 were generated using site-directed mutagenesis with Quickchange II (Agilent Technologies, Santa Clara, CA). The *vha-6p*::*gfp*::*glo-1*::*let-858 3’UTR* plasmid was used as the template for these reactions [1]. The primers P746 5’GGTGATCCAGGTGTCGGTAAAAACTCTATTATTCGTCG3’ and P747 5’CGACGAATAATAGAGTTTTTACCGACACCTGGATCACC3’ were used to generate GLO-1(T25N) and primers P744 ‘CTGGGATATTTCAGGCCTCGACCGATATGGGGTCATG3’ and P745 5’CATGACCCCATATCGGTCGAGGCCTGAAATATCCCAG3’ were used to generate GLO-1(Q71L). Underlined nucleotides denote the point mutations. The desired mutations, and lack of other mutations in the *glo-1* coding sequence, were confirmed by DNA sequencing. The 2.1kb *glo-1* promoter was added to *gfp*::*glo-1(+)*, *gfp*::*glo-1(T25N)*, *gfp*::*glo-1(Q71L)*, *gfp*::*glo-1(D132A)*, and *gfp*::*glo-1(I133F)* using PCR fusion [129]. The *glo-1* promoter was PCR amplified using genomic DNA as a template with primers P231 5’AACCCAAGCTTCCGTATCTTCTCTCCTTATTTCGACCG3’and P268 5’CAGTGAAAAGTTCTTCTCCTTTACTCATTTTGTTCTGAATATATATTAAAATTAG3’. *gfp*::*glo-1(*wild-type or mutant*)*::*let-858 3’UTR* was PCR amplified from plasmid templates using primers P269 5’ATGAGTAAAGGAGAAGAACTTTTCACTG3’and P500 5’ATTTCCCCGAAAAGTGCCACCTGACG3’. The promoter was added to the *glo-1* coding sequences with primers P265 5’ATAATGGGAACCTGAAATTAGAAGAGG3’ and P271 5’GACTAGTTTTCCTTCCTCCTCTATAT3’. The resulting fusion products were injected at 1ng/μl with the dominant *Rol-6* containing plasmid pRF4 at 100ng/μl. In all cases, multiple independent transgenic lines, which showed the same expression pattern, were isolated for each version of *glo-1*. Single arrays were chosen and crossed into different mutant backgrounds. In all studies with embryos, *glo-1* expression was controlled by its own promoter. Both the *glo-1* and *vha-6* promoters express in adult intestinal cells and thus in studies with adults, *glo-1* expression was controlled by either promoter.

## Supporting information

S1 Fig Gut granules in *glo-3(syb272)*. (A-B) Pretzel stage embryos were imaged with polarization microscopy. Birefringent gut granules present in wild type were lacking from the intestinal cells of *glo-3(syb272)* mutants and instead birefringent material accumulated in the intestinal lumen (black arrow). (C-D) Autofluorescent organelles present within adult intestinal cells were imaged with confocal microscopy. The number of autofluorescent gut granules was reduced in *glo-3(syb272)* mutants. The black arrows denote the location of the intestinal lumen. In A-D maximum intensity projections spanning the depth of the intestine are shown. (E-F) 1.5-fold stage embryos were stained with anti-PGP-2 and anti-LMP-1 antibodies and imaged with confocal microscopy. Both proteins colocalized at gut granules in wild type (white arrows within insets). *glo-3(syb272)* mutants lacked PGP-2 staining and LMP-1 accumulated on cytoplasmic organelles (white arrowheads within insets) and the cell membrane (black arrow). Single optical sections are shown. In A-B and E-F, the intestine is flanked by black arrowheads.
(TIF)

S2 Fig Activity of GLO-1(+). (A-F) 1.5-fold stage embryos expressing GFP::GLO-1(+) were stained with antibodies to PGP-2 and LMP-1. Embryos were imaged with confocal microscopy and single optical sections are shown. Black arrowheads flank the intestine and white arrows in the insets label organelles containing the gut granule protein PGP-2. The quantification of colocalization of LMP-1 or GLO-1::GFP with PGP-2 is shown in Fig 7.
(TIF)

S3 Fig The formation of the gut granule-like organelles in *glo-3(kx38)* mutants requires the function of gut granule biogenesis genes. (A-N) Signals from ectopically expressed CDF-2::GFP and antibodies to PGP-2 were acquired in 1.5-fold stage embryos using confocal microscopy. (A, G, H) In wild type, *sand-1*(-), and *cup-5(-)* mutants, CDF-2::GFP extensively colocalized with PGP-2 (white arrows in insets). (B, M, N) In *glo-3(kx38)* single and *glo-3(kx38); sand-1(-)* and *glo-3(kx38); cup-5(-)* double mutants, PGP-2 marked compartments contained CDF-2::GFP (white arrows in insets), however many CDF-2::GFP compartments lacked PGP-2 (black arrows in insets). (C-F) In gut granule biogenesis mutants, PGP-2 marked organelles were lacking and thus CDF-2::GFP containing compartments did not contain PGP-2 (black arrows in insets). (I-L) Similar effects on PGP-2 association with CDF-2::GFP marked organelles were seen when *glo-3(kx38)* was combined with these mutants. In A-N, black arrowheads flank the intestine. (O) SQUASSH software was used to calculate the area of PGP-2 organelles that contained CDF-2::GFP in strains that generated PGP-2 marked organelles. 5 embryos of each genotype were analyzed. The mean is plotted, bars represent the 95% confidence interval, and the double mutants were compared to *glo-3(kx38)* with a one way ANOVA, ns indicates p>0.05. The addition of *sand-1(-)* or *cup-5(-)* did not alter the localization of CDF-2::GFP to PGP-2 marked gut granules.
(TIF)

S4 Fig Characteristics of gut granules in *glo-3(kx38)* mutants. (A-C) Embryos expressing LMP-1::GFP, GFP::RAB-5, or GFP::RAB-7 were stained with anti-PGP-2 antibodies and imaged with wide-field fluorescence microscopy. (A) LMP-1::GFP did not accumulate on PGP-2 compartments in wild-type or *glo-3(kx38)* 1.5-fold stage embryos (white arrowheads in insets). (B) In pretzel stage embryos, GFP::RAB-5 did not associate with PGP-2 marked organelles (white arrows in insets). (C) While GFP::RAB-7 was not enriched on PGP-2 containing gut granules in wild-type pretzel stage embryos (white arrowheads in insets) it accumulated on PGP-2 organelles in *glo-3(kx38)* mutants (arrows in insets). (D-E) In living 1.5-fold stage embryos neither lysosomal hydrolase, CPR-6::mCherry or GBA-3::mCherry, associated with autofluorescent gut granules in wild type or *glo-3(kx38)* mutants (white arrowheads in insets). Embryos were visualized with wide-field fluorescence microscopy. (F-I) Embryos expressing ARL-8::GFP, GFP::RAB-10, GFP::RAB-11.1, or MANS::GFP were stained with anti-PGP-2 antibodies and imaged with confocal microscopy. (F) ARL-8::GFP did not accumulate on PGP-2 compartments in wild-type or *glo-3(kx38)* 1.5-fold stage embryos (white arrowheads in insets). (G-I) In wild-type and *glo-3(kx38)* pretzel stage embryos, GFP::RAB-10, GFP::RAB-11.1, and MANS::GFP did not associate with PGP-2 marked organelles (white arrows in insets). In A-I, the intestine is flanked by black arrowheads. (J) Colocalization was scored in 3–5 embryos of each genotype by randomly selecting 20 PGP-2 or autofluorescent organelles and assessing for the presence of the GFP or mCherry signals. *glo-3(kx38)* mutants have reduced numbers of gut granules (typically between 15–20) all of which were scored. The mean is plotted and bars represent the 95% confidence intervals and ** indicates p≤0.001 by one way ANOVA comparing *glo-3(kx38)* to wild type.
(TIF)

S5 Fig RAB-7 is mislocalized to, and LMP-1 is lacking from, gut granules in the *glo-3(gk582755)* class IV mutant. (A and C) Signals from antibodies to PGP-2 and RAB-7 or LMP-1 were acquired in 1.5-fold stage embryos using confocal microscopy. (A) In wild type, RAB-7 did not associate with gut granules (black arrows in insets). However, in *glo-3(gk582755)* mutants, RAB-7 mislocalized to PGP-2 marked gut granules (white arrows in insets). (C) While PGP-2 compartments contained LMP-1 in wild type (white arrows in insets), the anti-LMP-1 signals were weak or lacking from gut granules in *glo-3(gk582755)* mutants (black arrows in insets). (B and D) Colocalization was scored by randomly selecting 40 PGP-2 marked organelles in wild type or all of the PGP-2 marked organelles (30–40) in *glo-3(gk582755)* embryos and assessing the presence of RAB-7 or LMP-1 signal. Five embryos were scored for each genotype. The mean is plotted and bars represent the 95% confidence interval and ** indicates p≤0.001 by one way ANOVA comparing *glo-3(gk582755)* to wild type.
(TIF)

S6 Fig RAB-7 and LMP-1 colocalization with gut granules in the *tbc-2(tm2241)* mutant. (A and C) Signals from antibodies to PGP-2 and RAB-7 or LMP-1 were acquired in 1.5-fold stage embryos using confocal microscopy. (A) In wild type and *tbc-2(tm2241)*, RAB-7 did not associate with gut granules (white arrows in insets). (C) PGP-2 compartments contained LMP-1 in wild type and *tbc-2(tm2241)* mutants (black arrows in insets). (B and D) Colocalization was scored by randomly selecting 20–30 PGP-2 marked organelles in five 1.5-fold stage embryos and assessing the presence of RAB-7 or LMP-1 signals. Five embryos were scored for each genotype. The mean is plotted and bars represent the 95% confidence interval. ** indicates p≤0.001 by one way ANOVA comparing *tbc-2(tm2241)* to wild type.
(TIF)

S1 Supporting Numerical Data This spreadsheet contains all of the numerical data that underlies the graphs in the manuscript. (XLSX)
