# Supplementary figures and images for: Function and regulation of the Caenorhabditis elegans Rab32 family member GLO-1 in lysosome-related organelle biogenesis
Source: PLoS Genet. 2018 Nov 12;14(11):e1007772. doi: 10.1371/journal.pgen.1007772 (PMC6268011; doi:10.1371/journal.pgen.1007772)

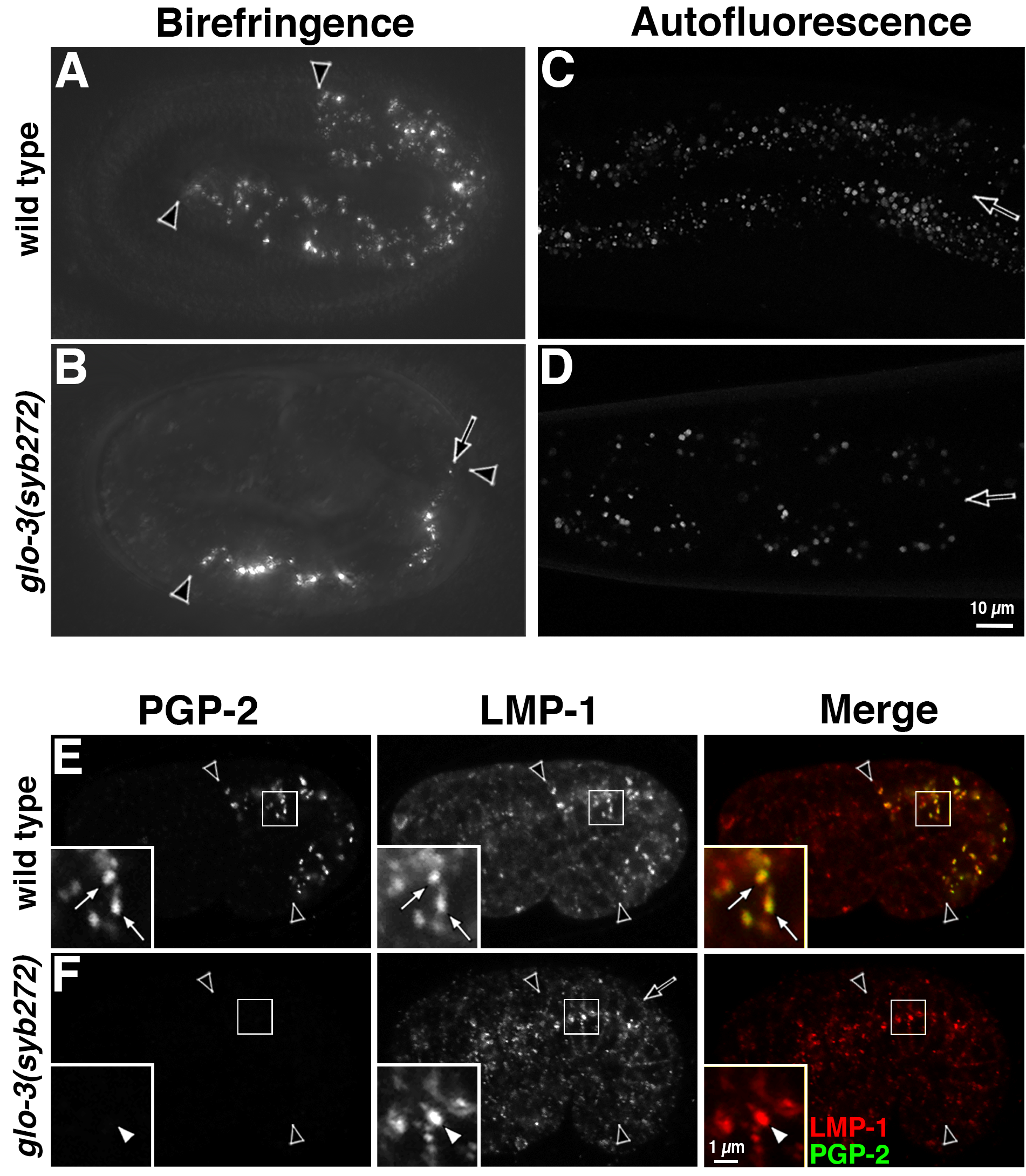

Supplement: S1 Fig — (A-B) Pretzel stage embryos were imaged with polarization microscopy. Birefringent gut granules present in wild type were lacking from the intestinal cells of glo-3(syb272) mutants and instead birefringent material accumulated in the intestinal lumen (black arrow). (C-D) Autofluorescent organelles present within adult intestinal cells were imaged with confocal microscopy. The number of autofluorescent gut granules was reduced in glo-3(syb272) mutants. The black arrows denote the location of the intestinal lumen. In A-D maximum intensity projections spanning the depth of the intestine are shown. (E-F) 1.5-fold stage embryos were stained with anti-PGP-2 and anti-LMP-1 antibodies and imaged with confocal microscopy. Both proteins colocalized at gut granules in wild type (white arrows within insets). glo-3(syb272) mutants lacked PGP-2 staining and LMP-1 accumulated on cytoplasmic organelles (white arrowheads within insets) and the cell membrane (black arrow). Single optical sections are shown. In A-B and E-F, the intestine is flanked by black arrowheads. (TIF) [file pgen.1007772.s001.tif]

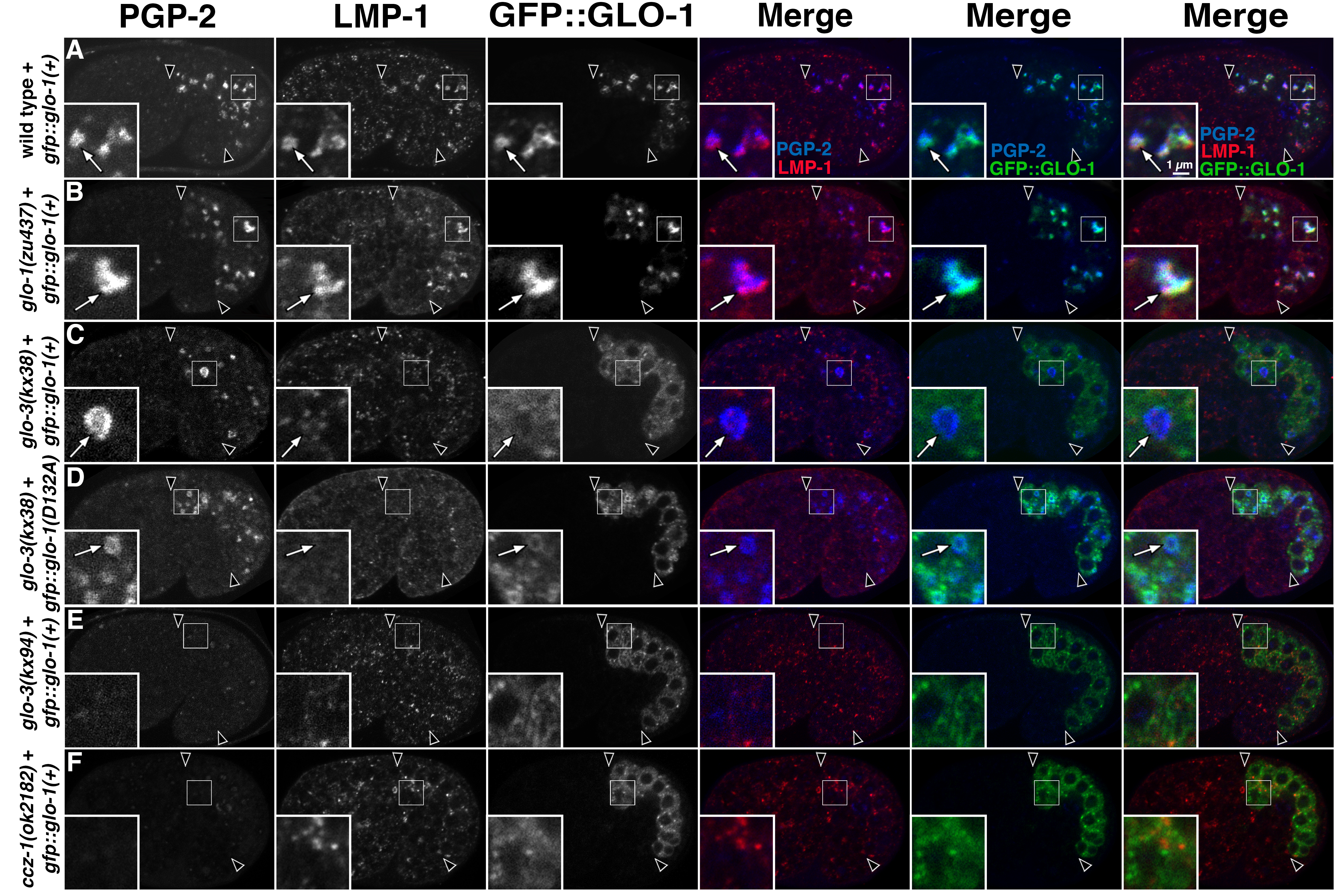

Supplement: S2 Fig — (A-F) 1.5-fold stage embryos expressing GFP::GLO-1(+) were stained with antibodies to PGP-2 and LMP-1. Embryos were imaged with confocal microscopy and single optical sections are shown. Black arrowheads flank the intestine and white arrows in the insets label organelles containing the gut granule protein PGP-2. The quantification of colocalization of LMP-1 or GLO-1::GFP with PGP-2 is shown in Fig 7. (TIF) [file pgen.1007772.s002.tif]

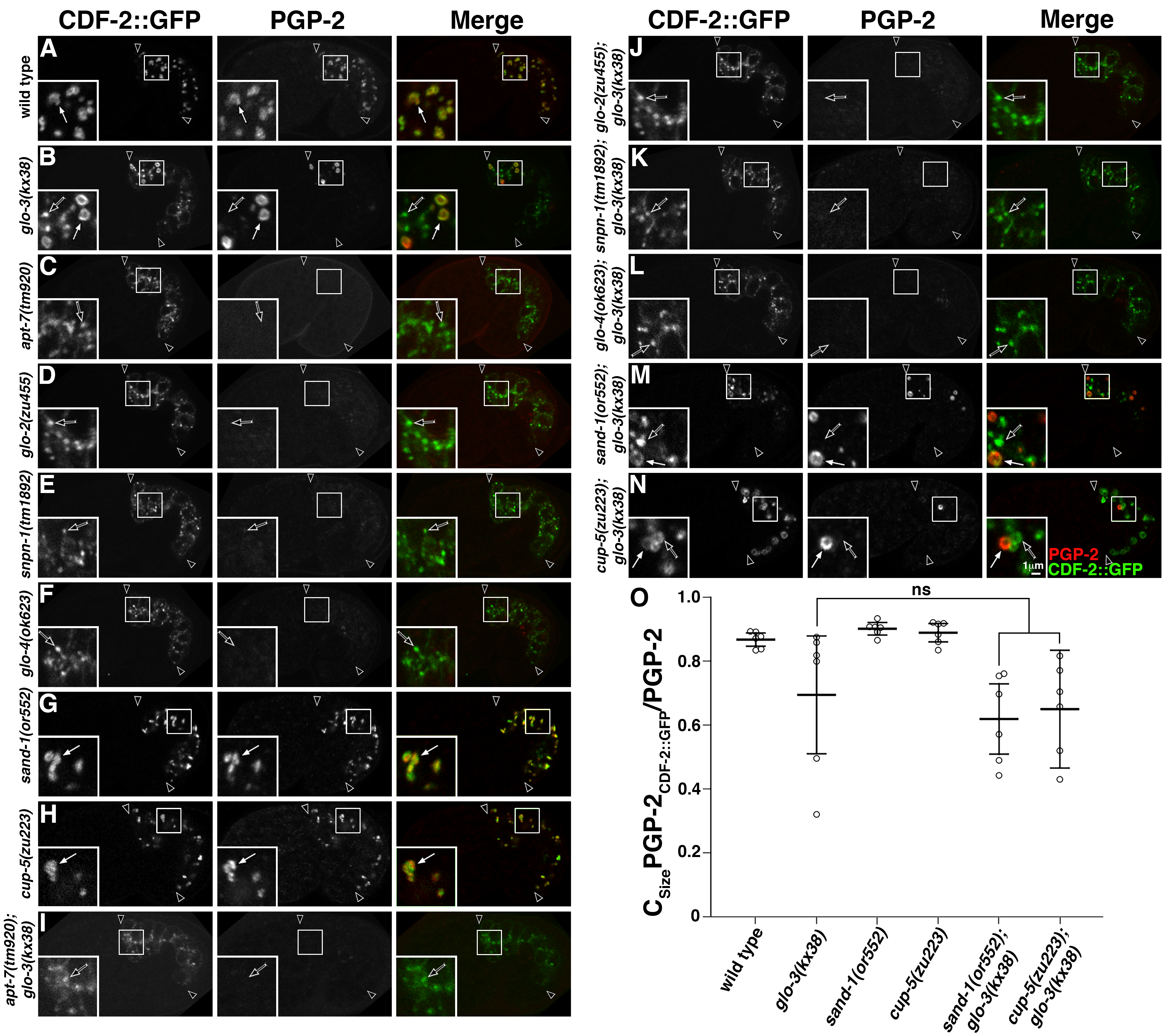

Supplement: S3 Fig — (A-N) Signals from ectopically expressed CDF-2::GFP and antibodies to PGP-2 were acquired in 1.5-fold stage embryos using confocal microscopy. (A, G, H) In wild type, sand-1(-), and cup-5(-) mutants, CDF-2::GFP extensively colocalized with PGP-2 (white arrows in insets). (B, M, N) In glo-3(kx38) single and glo-3(kx38); sand-1(-) and glo-3(kx38); cup-5(-) double mutants, PGP-2 marked compartments contained CDF-2::GFP (white arrows in insets), however many CDF-2::GFP compartments lacked PGP-2 (black arrows in insets). (C-F) In gut granule biogenesis mutants, PGP-2 marked organelles were lacking and thus CDF-2::GFP containing compartments did not contain PGP-2 (black arrows in insets). (I-L) Similar effects on PGP-2 association with CDF-2::GFP marked organelles were seen when glo-3(kx38) was combined with these mutants. In A-N, black arrowheads flank the intestine. (O) SQUASSH software was used to calculate the area of PGP-2 organelles that contained CDF-2::GFP in strains that generated PGP-2 marked organelles. 5 embryos of each genotype were analyzed. The mean is plotted, bars represent the 95% confidence interval, and the double mutants were compared to glo-3(kx38) with a one way ANOVA, ns indicates p>0.05. The addition of sand-1(-) or cup-5(-) did not alter the localization of CDF-2::GFP to PGP-2 marked gut granules. (TIF) [file pgen.1007772.s003.tif]

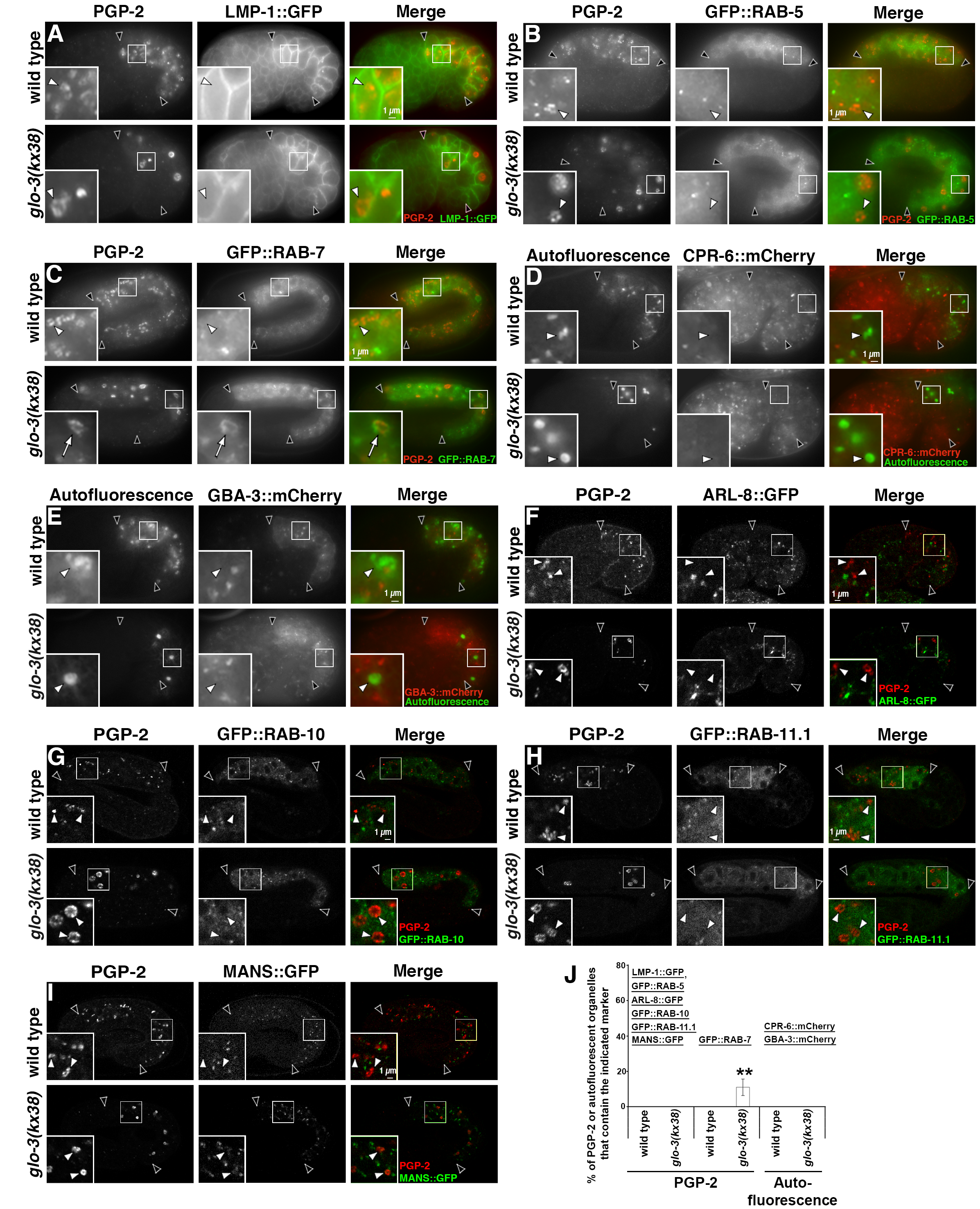

Supplement: S4 Fig — (A-C) Embryos expressing LMP-1::GFP, GFP::RAB-5, or GFP::RAB-7 were stained with anti-PGP-2 antibodies and imaged with wide-field fluorescence microscopy. (A) LMP-1::GFP did not accumulate on PGP-2 compartments in wild-type or glo-3(kx38) 1.5-fold stage embryos (white arrowheads in insets). (B) In pretzel stage embryos, GFP::RAB-5 did not associate with PGP-2 marked organelles (white arrows in insets). (C) While GFP::RAB-7 was not enriched on PGP-2 containing gut granules in wild-type pretzel stage embryos (white arrowheads in insets) it accumulated on PGP-2 organelles in glo-3(kx38) mutants (arrows in insets). (D-E) In living 1.5-fold stage embryos neither lysosomal hydrolase, CPR-6::mCherry or GBA-3::mCherry, associated with autofluorescent gut granules in wild type or glo-3(kx38) mutants (white arrowheads in insets). Embryos were visualized with wide-field fluorescence microscopy. (F-I) Embryos expressing ARL-8::GFP, GFP::RAB-10, GFP::RAB-11.1, or MANS::GFP were stained with anti-PGP-2 antibodies and imaged with confocal microscopy. (F) ARL-8::GFP did not accumulate on PGP-2 compartments in wild-type or glo-3(kx38) 1.5-fold stage embryos (white arrowheads in insets). (G-I) In wild-type and glo-3(kx38) pretzel stage embryos, GFP::RAB-10, GFP::RAB-11.1, and MANS::GFP did not associate with PGP-2 marked organelles (white arrows in insets). In A-I, the intestine is flanked by black arrowheads. (J) Colocalization was scored in 3–5 embryos of each genotype by randomly selecting 20 PGP-2 or autofluorescent organelles and assessing for the presence of the GFP or mCherry signals. glo-3(kx38) mutants have reduced numbers of gut granules (typically between 15–20) all of which were scored. The mean is plotted and bars represent the 95% confidence intervals and ** indicates p≤0.001 by one way ANOVA comparing glo-3(kx38) to wild type. (TIF) [file pgen.1007772.s004.tif]

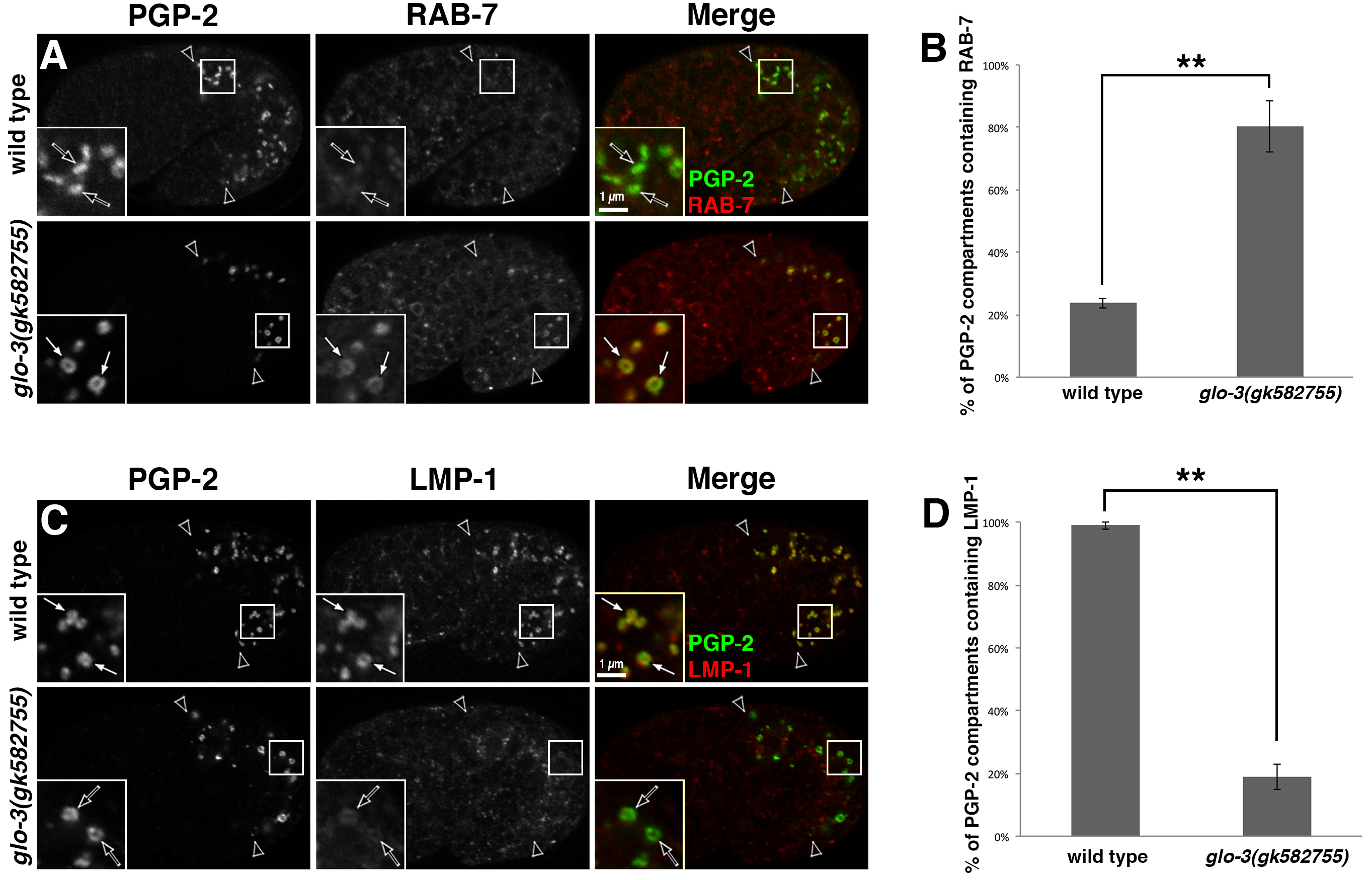

Supplement: S5 Fig — (A and C) Signals from antibodies to PGP-2 and RAB-7 or LMP-1 were acquired in 1.5-fold stage embryos using confocal microscopy. (A) In wild type, RAB-7 did not associate with gut granules (black arrows in insets). However, in glo-3(gk582755) mutants, RAB-7 mislocalized to PGP-2 marked gut granules (white arrows in insets). (C) While PGP-2 compartments contained LMP-1 in wild type (white arrows in insets), the anti-LMP-1 signals were weak or lacking from gut granules in glo-3(gk582755) mutants (black arrows in insets). (B and D) Colocalization was scored by randomly selecting 40 PGP-2 marked organelles in wild type or all of the PGP-2 marked organelles (30–40) in glo-3(gk582755) embryos and assessing the presence of RAB-7 or LMP-1 signal. Five embryos were scored for each genotype. The mean is plotted and bars represent the 95% confidence interval and ** indicates p≤0.001 by one way ANOVA comparing glo-3(gk582755) to wild type. (TIF) [file pgen.1007772.s005.tif]

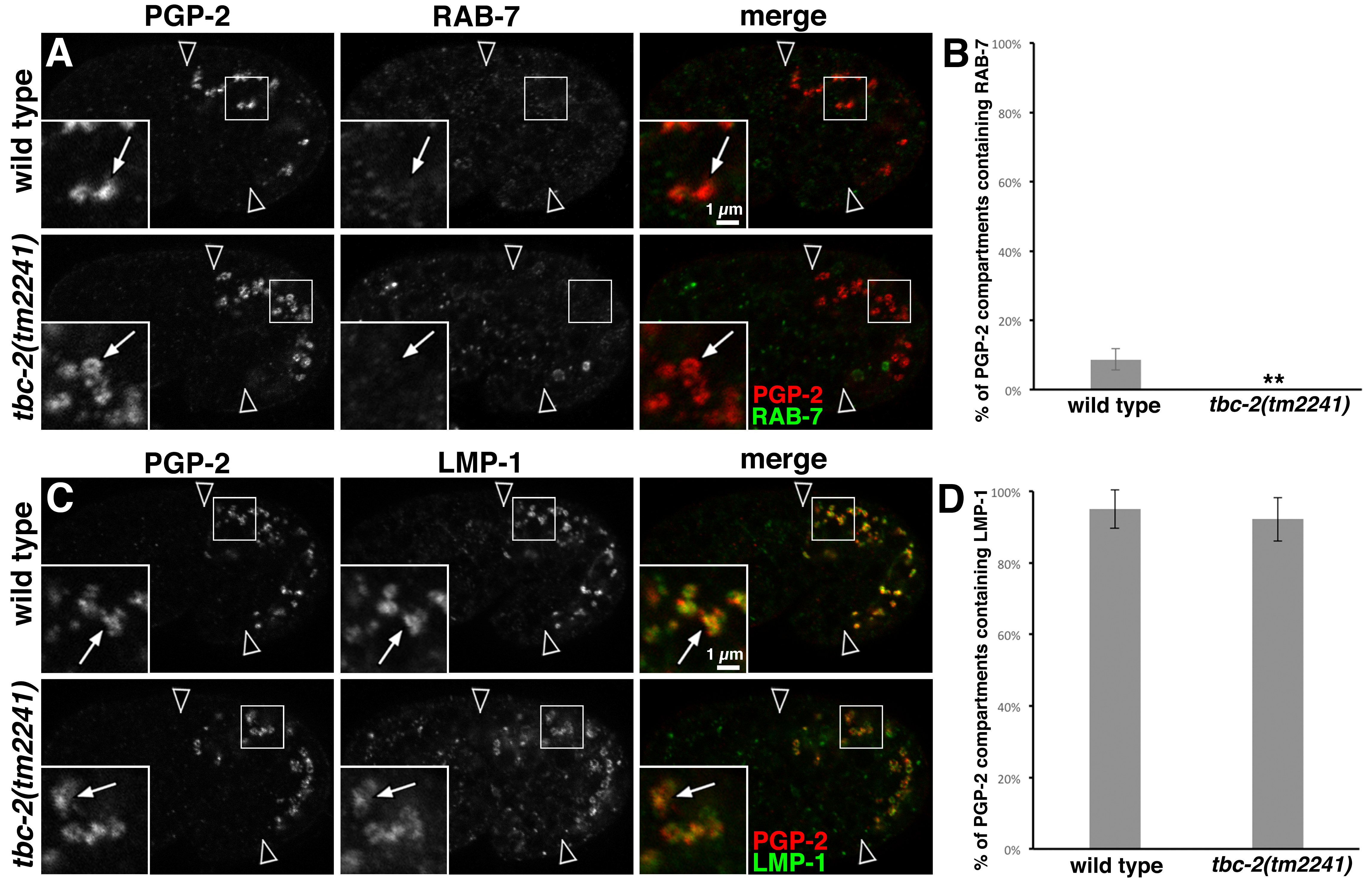

Supplement: S6 Fig — (A and C) Signals from antibodies to PGP-2 and RAB-7 or LMP-1 were acquired in 1.5-fold stage embryos using confocal microscopy. (A) In wild type and tbc-2(tm2241), RAB-7 did not associate with gut granules (white arrows in insets). (C) PGP-2 compartments contained LMP-1 in wild type and tbc-2(tm2241) mutants (black arrows in insets). (B and D) Colocalization was scored by randomly selecting 20–30 PGP-2 marked organelles in five 1.5-fold stage embryos and assessing the presence of RAB-7 or LMP-1 signals. Five embryos were scored for each genotype. The mean is plotted and bars represent the 95% confidence interval. ** indicates p≤0.001 by one way ANOVA comparing tbc-2(tm2241) to wild type. (TIF) [file pgen.1007772.s006.tif]
